# Supplementary material for: Induction chemotherapy plus camrelizumab followed by concurrent chemoradiotherapy in unresectable locally advanced esophageal squamous cell carcinoma: a single-arm phase II trial
Source: Nat Commun. 2025 Nov 21;16:10292. doi: 10.1038/s41467-025-65206-z (PMC12638862; doi:10.1038/s41467-025-65206-z)
Supplement: Supplementary file 1 — Supplementary Information [file 41467_2025_65206_MOESM1_ESM.pdf]

SUPPLEMENTARY INFORMATION

Induction chemotherapy plus camrelizumab followed by concurrent chemoradiotherapy in unresectable locally advanced esophageal squamous cell carcinoma: a single-arm phase II trial

Fang Peng[1]†, Jialiang Wu[2]†, Huimin Lian[1], Shuang Wu[1], Shaoqing Niu[1], Xiangbin Xing[3], Weixiong Yang[4], Wu Song[5], Yin Li[5], Honglan Yu[5], Shi-Ting Feng[6], Xiaoyan Wang[7], Wenfang Chen[8], Wen Ye[9], Tiantian Yu[1], Weijian Liufu[1], Chao Cheng[4]*, Yong Bao[1]*

Correspondence to: Chao Cheng (chengch3@mail.sysu.edu.cn), Yong Bao (baoyong@mail.sysu.edu.cn)

This PDF file includes:
Supplementary Figure 1 to 17
Supplementary Table 1 to 4
Supplementary Note. Clinical Research Protocol

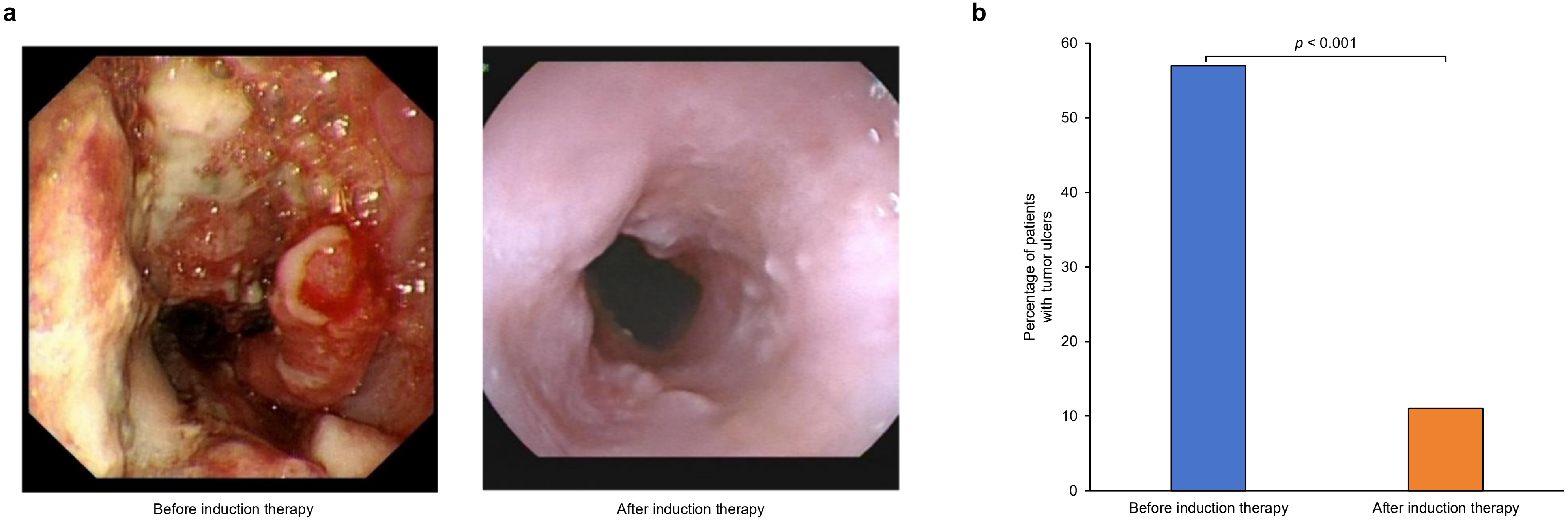

Supplementary Figure 1.

Changes in tumor ulceration before and after induction chemotherapy plus camrelizumab. **(a)** Representative endoscopic images demonstrating healing of tumor ulcers following induction chemotherapy plus camrelizumab. **(b)** Incidence of tumor ulcers before and after induction therapy (n = 46). Statistical analysis was performed using McNemar's test (two-sided). $P < 0.05$ was considered statistically significant. Source data are provided as a Source Data file.

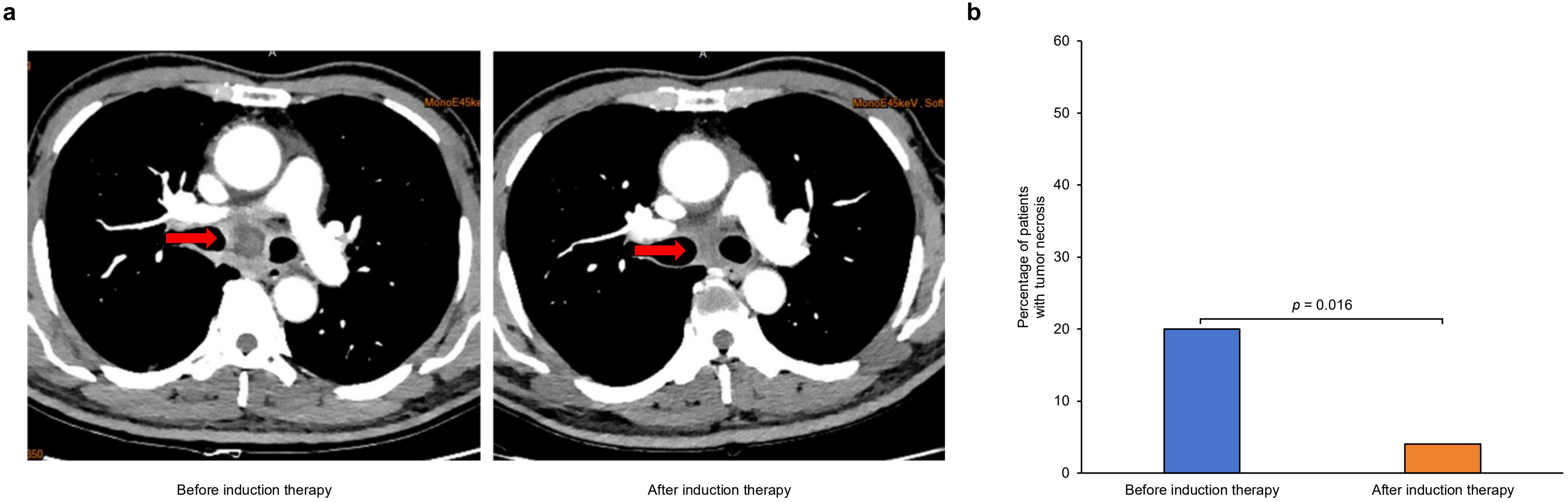

Supplementary Figure 2.

Changes in tumor necrosis before and after induction chemotherapy plus camrelizumab. **(a)** Enhanced CT images of a representative patient demonstrating significant improvement in tumor necrosis following induction chemotherapy plus camrelizumab. **(b)** Incidence of tumor necrosis before and after induction therapy (n = 46). Statistical analysis was performed using McNemar's test (two-sided). $P < 0.05$ was considered statistically significant. Source data are provided as a Source Data file.

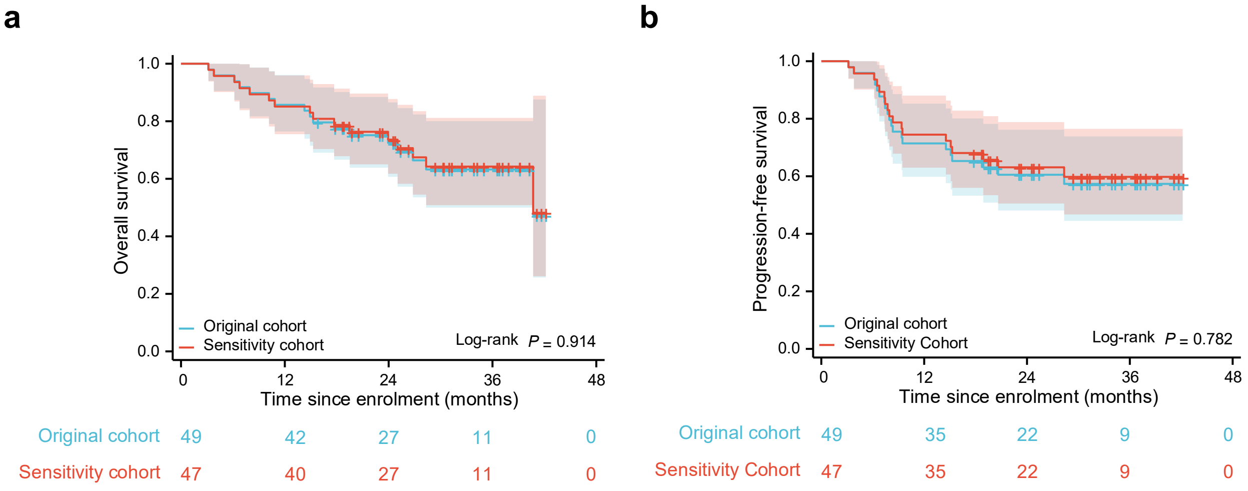

Supplementary Figure 3.

Sensitivity analysis comparing the original cohort and the sensitivity cohort (excluding two patients who experienced radiotherapy interruptions due to COVID-19). **(a)** Overall survival. **(b)** Progression-free survival. Kaplan-Meier survival curves are shown with 95% confidence intervals (shaded areas). Statistical analysis was performed using the log-rank test (two-sided). $P < 0.05$ was considered statistically significant. Source data are provided as a Source Data file.

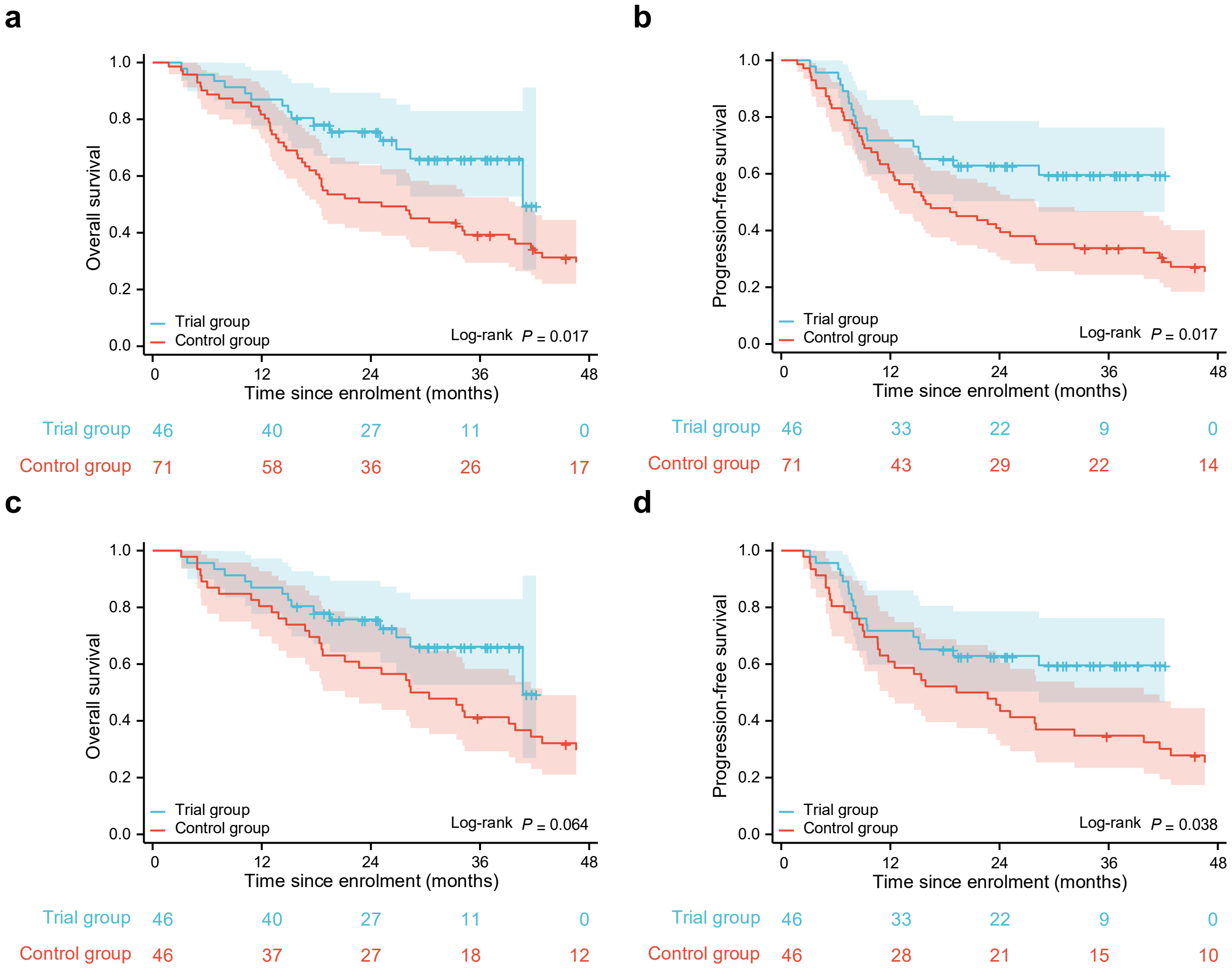

Supplementary Figure 4.

Survival comparison between the trial group and control group (historical control). **(a)** Overall survival. **(b)** Progression-free survival. **(c)** Overall survival after PSM. **(d)** Progression-free survival after PSM. Kaplan-Meier survival curves are shown with 95% confidence intervals (shaded areas). Statistical analysis was performed using the log-rank test (two-sided). $P < 0.05$ was considered statistically significant. Source data are provided as a Source Data file. PSM propensity score matching.

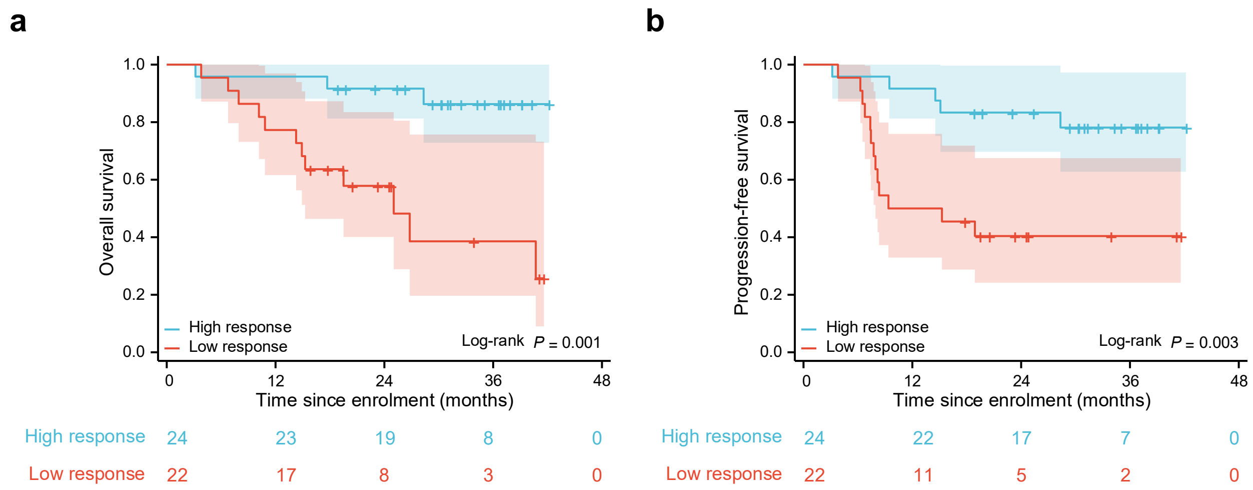

Supplementary Figure 5.

The effect of tumor response to induction chemotherapy plus camrelizumab on survival outcome. **(a)** Overall survival. **(b)** Progression-free survival. Kaplan-Meier survival curves are shown with 95% confidence intervals (shaded areas). Statistical analysis was performed using the log-rank test (two-sided). $P < 0.05$ was considered statistically significant. Source data are provided as a Source Data file.

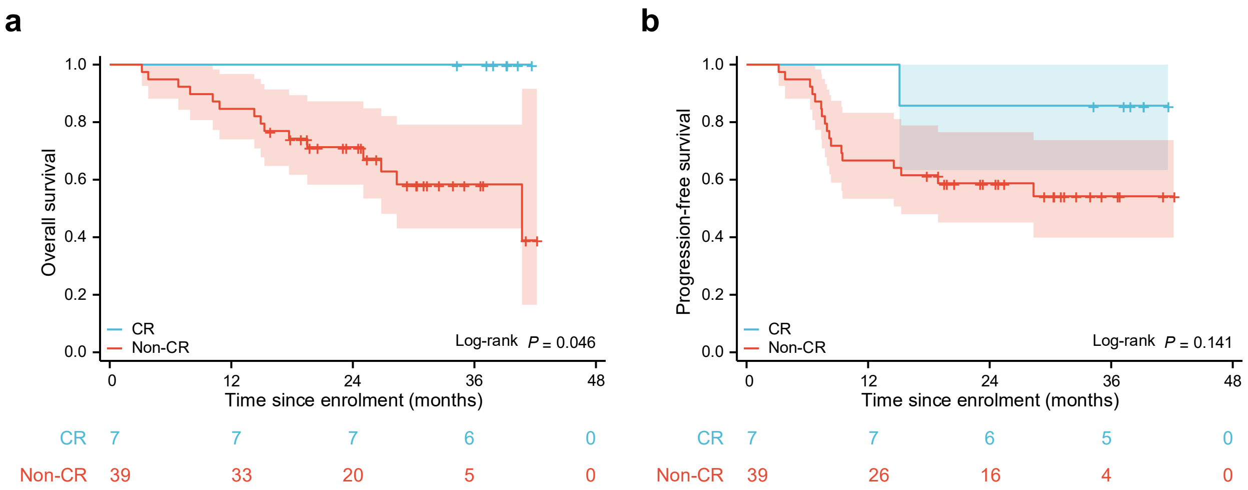

Supplementary Figure 6.

Survival comparison between patients achieving CR after induction chemotherapy plus camrelizumab followed by concurrent chemoradiotherapy and those without CR. **(a)** Overall survival. **(b)** Progression-free survival. Kaplan-Meier survival curves are shown with 95% confidence intervals (shaded areas). Statistical analysis was performed using the log-rank test (two-sided). $P < 0.05$ was considered statistically significant. Source data are provided as a Source Data file. CR complete response.

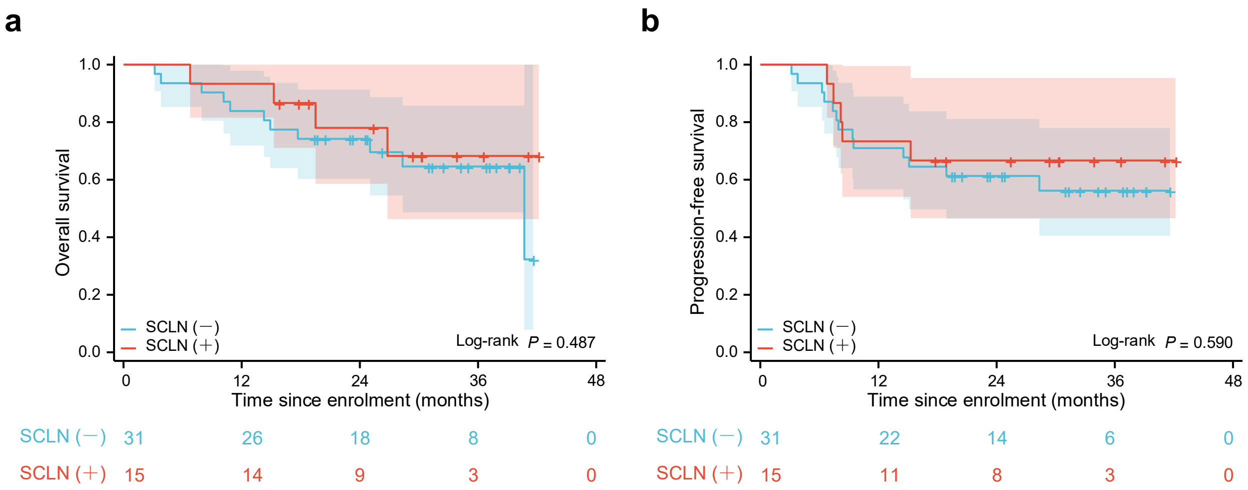

Supplementary Figure 7.

Survival comparison between patients without SCLN metastasis and those with SCLN metastasis. **(a)** Overall survival. **(b)** Progression-free survival. Kaplan-Meier survival curves are shown with 95% confidence intervals (shaded areas). Statistical analysis was performed using the log-rank test (two-sided). $P < 0.05$ was considered statistically significant. Source data are provided as a Source Data file. SCLN supraclavicular lymph node.

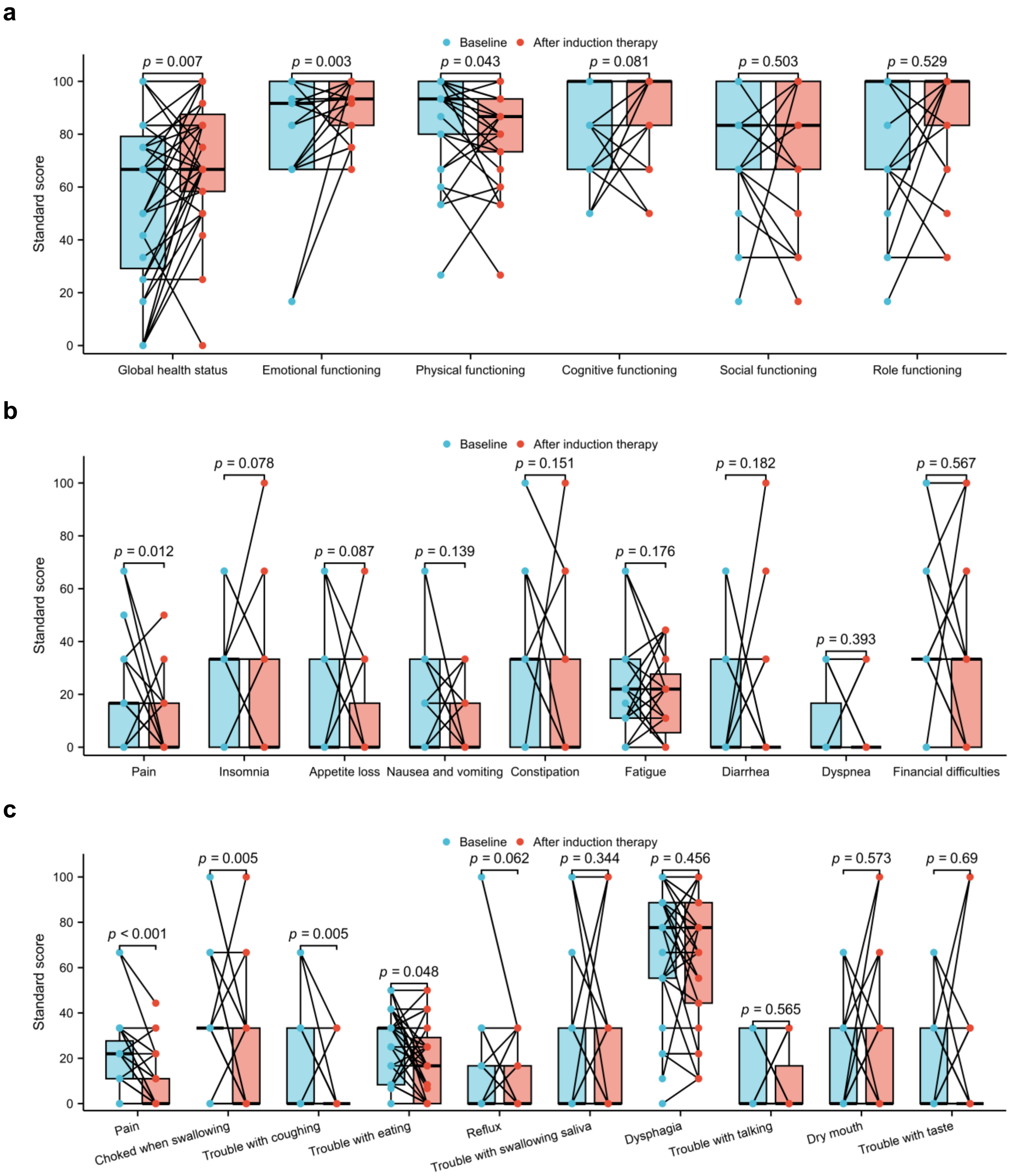

Supplementary Figure 8.

Changes in health-related quality of life between baseline and post-induction therapy using the QLQ-C30 and QLQ-OES18 scales. **(a)** Global health status and function (n = 35). **(b)** Constitutional cancer symptoms (n = 35). **(c)** Esophageal cancer specific symptoms (n = 39). Box plots show median (center line), first and third quartiles (box edges), and 1.5× interquartile range (whiskers). Individual data points are shown as dots, with connecting lines indicating paired observations. Dots beyond whiskers represent outliers. Statistical analysis was performed using the Wilcoxon signed-rank test (two-sided). $P < 0.05$ was considered statistically significant. Source data are provided as a Source Data file. QLQ-C30 Quality of Life Questionnaire-Core 30, QLQ-OES18 Quality of Life Questionnaire-Esophageal Cancer Module-18.

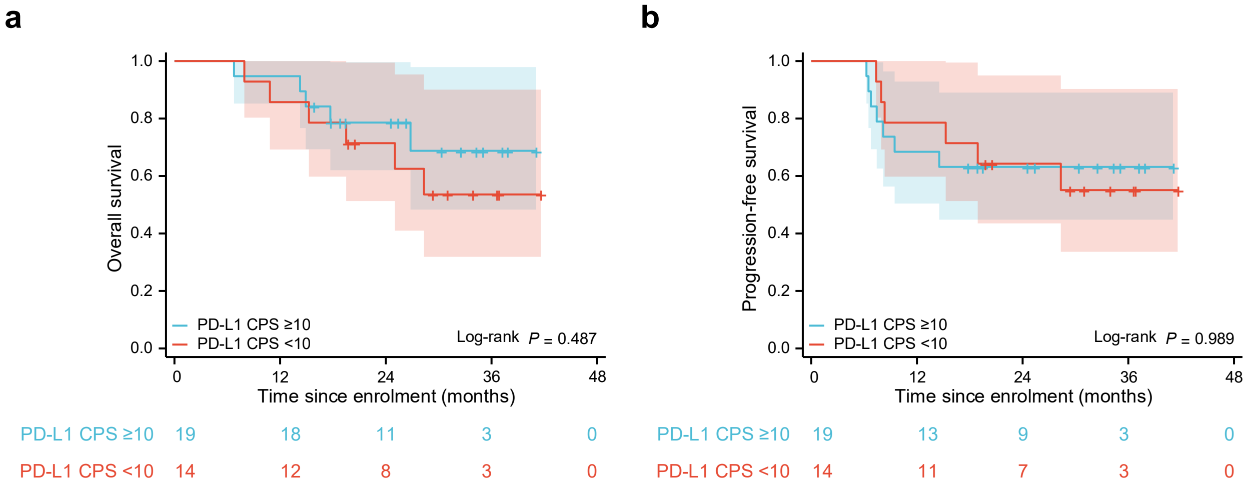

Supplementary Figure 9.

The effect of PD-L1 expression on survival outcome. **(a)** Overall survival. **(b)** Progression-free survival. Kaplan-Meier survival curves are shown with 95% confidence intervals (shaded areas). Statistical analysis was performed using the log-rank test (two-sided). $P < 0.05$ was considered statistically significant. Source data are provided as a Source Data file. PD-L1 programmed cell death-ligand 1, CPS combined positivity score.

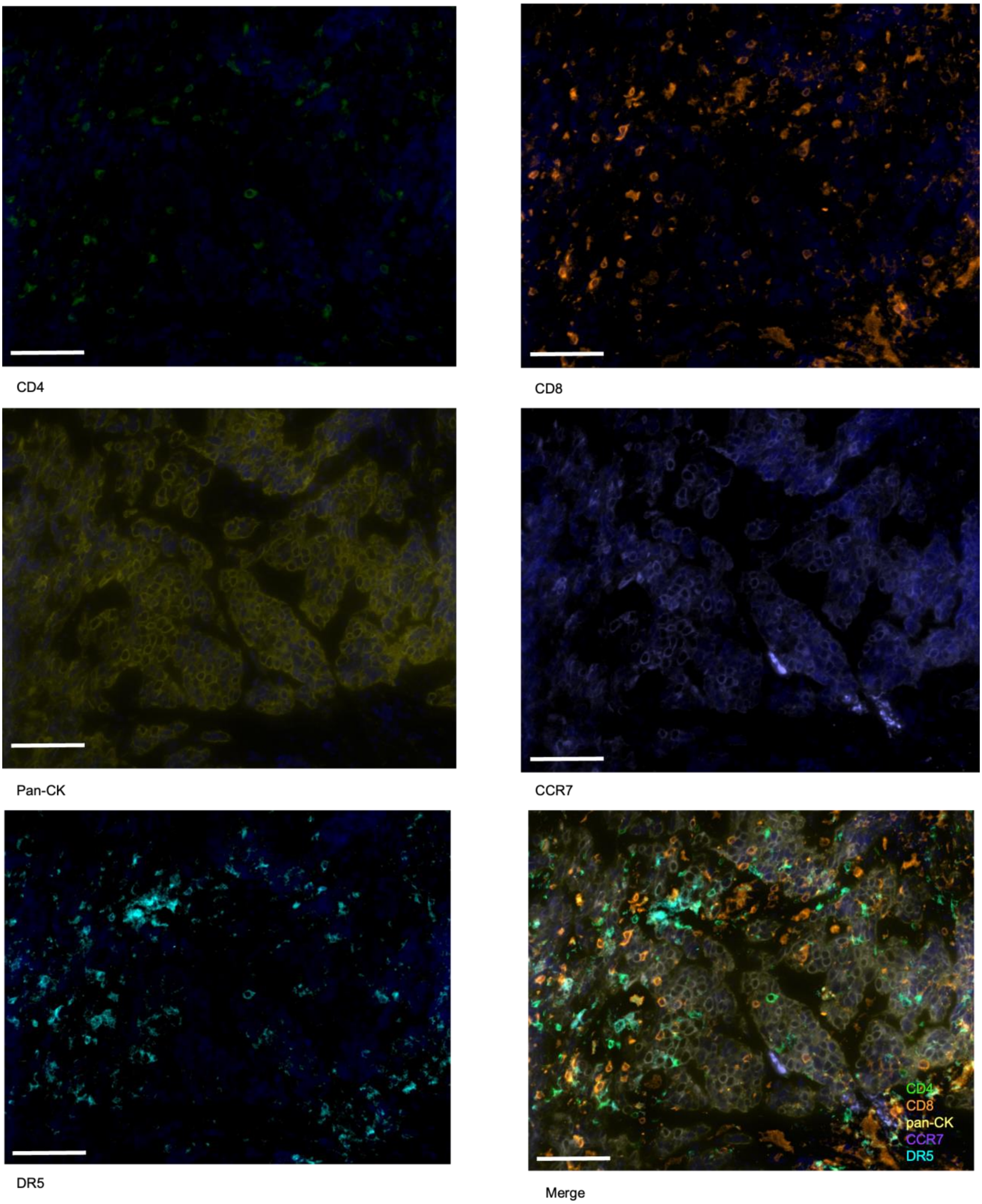

Supplementary Figure 10.

Multiplex immunofluorescence staining for CD4, CD8, pan-CK, CCR7, and DR5 in a high responder. Scale bars: 100 μm. CD4 cluster of differentiation 4, CD8 cluster of differentiation 8, pan-CK pan-cytokeratin, CCR7 C-C chemokine receptor 7, DR5 death receptor 5.

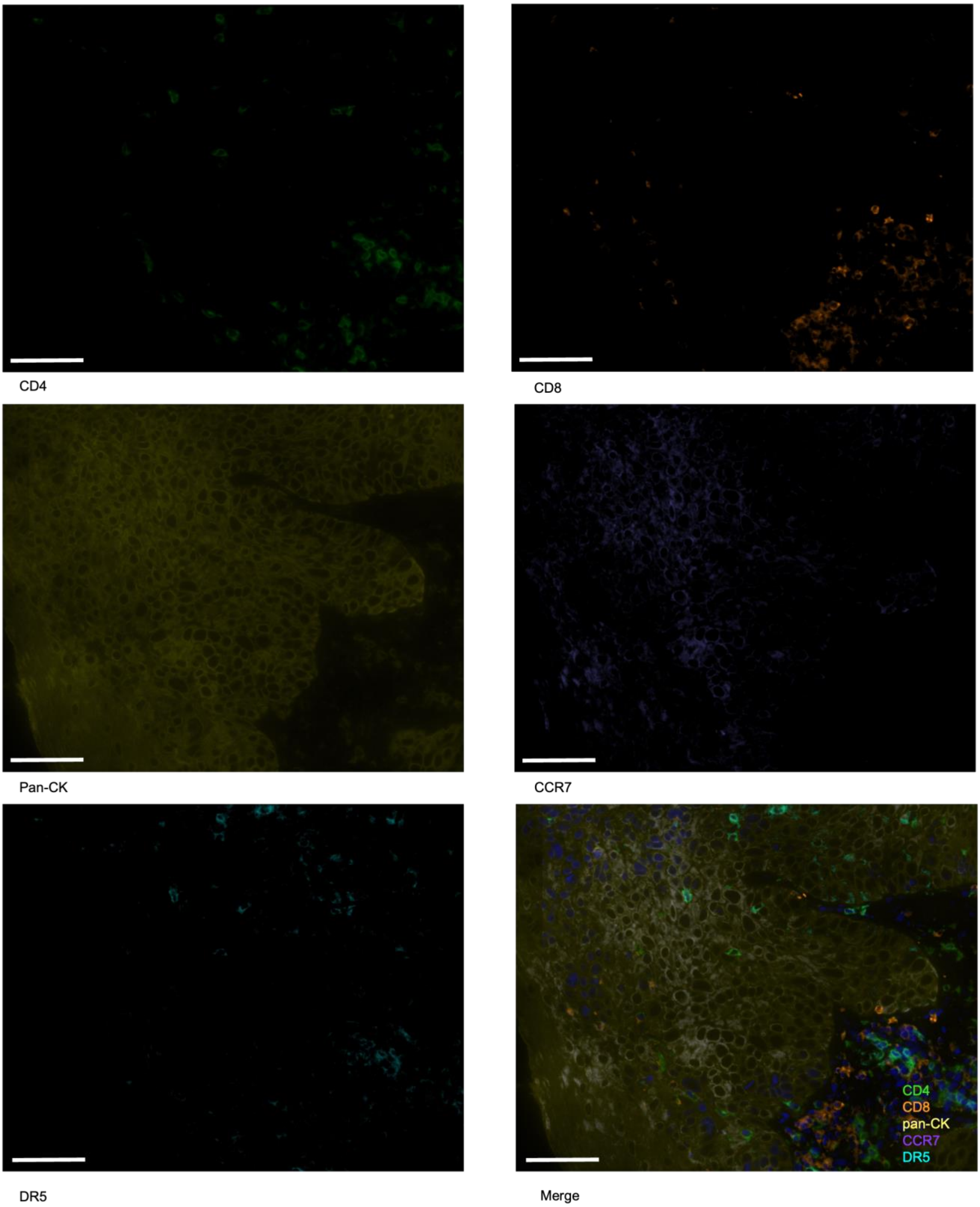

Supplementary Figure 11.

Multiplex immunofluorescence staining for CD4, CD8, pan-CK, CCR7, and DR5 in a low responder. Scale bars: 100 μm. CD4 cluster of differentiation 4, CD8 cluster of differentiation 8, pan-CK pan cytokeratin, CCR7 C-C chemokine receptor 7, DR5 death receptor 5.

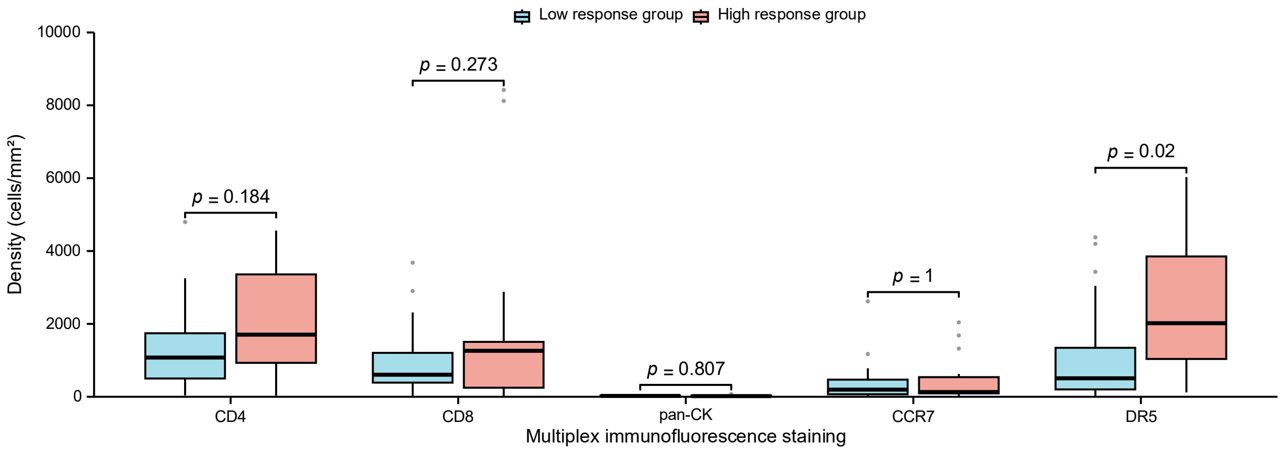

Supplementary Figure 12.

Relationship of tumour response with immune cell infiltration (n = 35). Box plots show median (center line), first and third quartiles (box edges), and 1.5× interquartile range (whiskers). Dots beyond whiskers represent outliers. Statistical analysis was performed using the Mann-Whitney U test (two-sided). $P < 0.05$ was considered statistically significant. Source data are provided as a Source Data file. CD4 cluster of differentiation 4, CD8 cluster of differentiation 8, pan-CK pan-cytokeratin, CCR7 C-C chemokine receptor 7, DR5 death receptor 5.

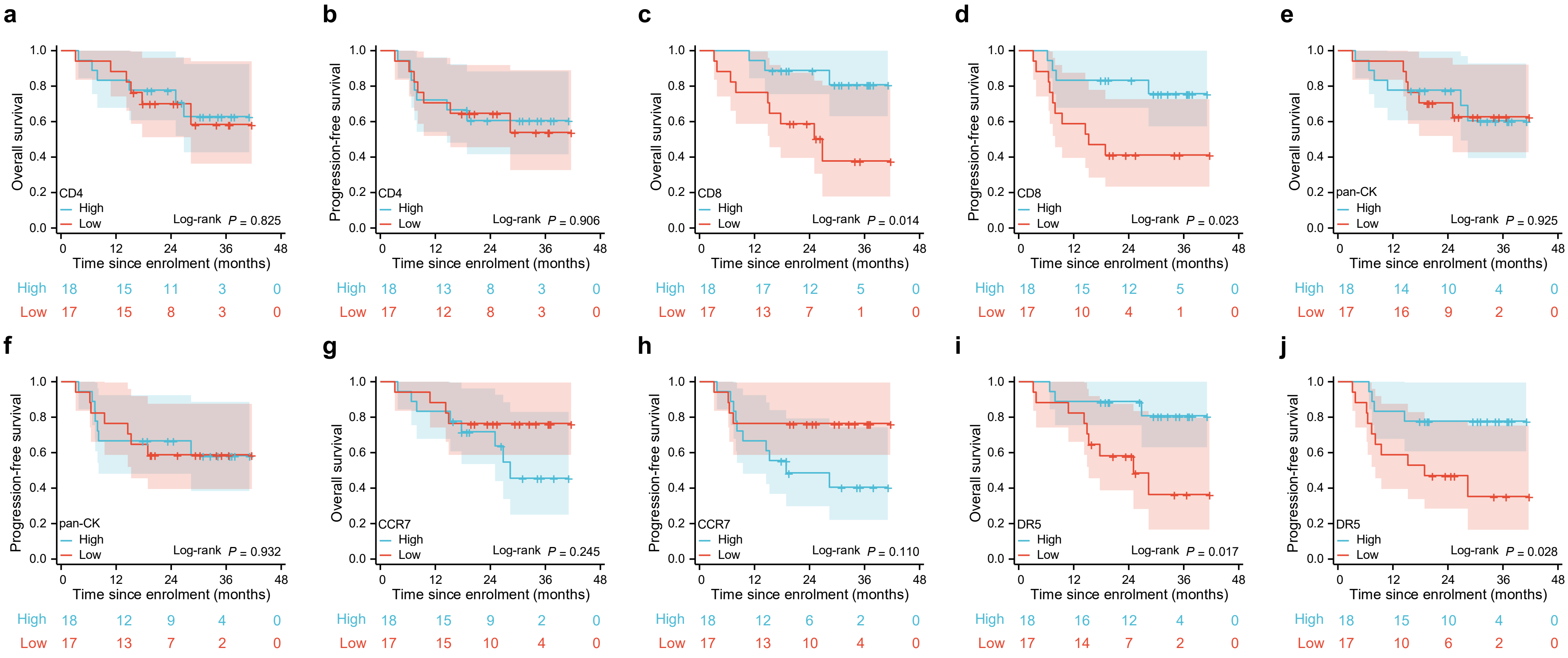

Supplementary Figure 13.

Comparison of treatment outcomes between patients with low expression and high expression of different immune cell infiltration using multiplex immunofluorescence staining. **(a)** Relationship of overall survival (OS) with CD4 density. **(b)** Relationship of progression-free survival (PFS) with CD4 density. **(c)** Relationship of OS with CD8 density. **(d)** Relationship of PFS with CD8 density. **(e)** Relationship of OS with pan-CK density. **(f)** Relationship of PFS with pan-CK density. **(g)** Relationship of OS with CCR7 density. **(h)** Relationship of PFS with CCR7 density. **(i)** Relationship of OS with DR5 density. **(j)** Relationship of PFS with DR5 density. Kaplan-Meier survival curves are shown with 95% confidence intervals (shaded areas). Statistical analysis was performed using the log-rank test (two-sided). $P < 0.05$ was considered statistically significant. Source data are provided as a Source Data file. CD4 cluster of differentiation 4, CD8 cluster of differentiation 8, pan-CK pan-cytokeratin, CCR7 C-C chemokine receptor 7, DR5 death receptor 5.

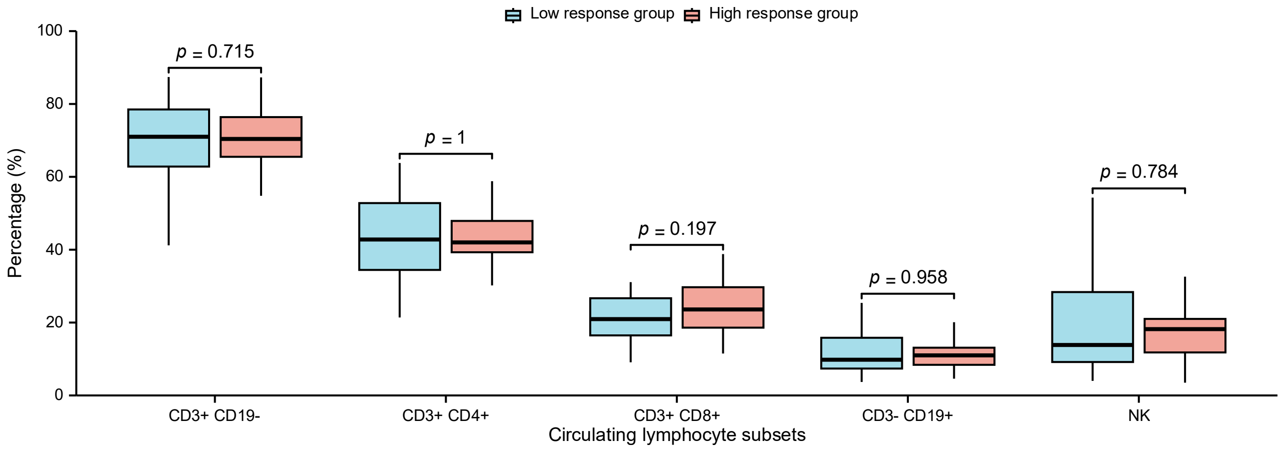

Supplementary Figure 14.

Relationship of tumour response with lymphocyte subsets in peripheral blood (n = 41). Box plots show median (center line), first and third quartiles (box edges), and 1.5× interquartile range (whiskers). Dots beyond whiskers represent outliers. Statistical analysis was performed using the Mann-Whitney U test (two-sided). *P* < 0.05 was considered statistically significant. Source data are provided as a Source Data file. NK natural killer.

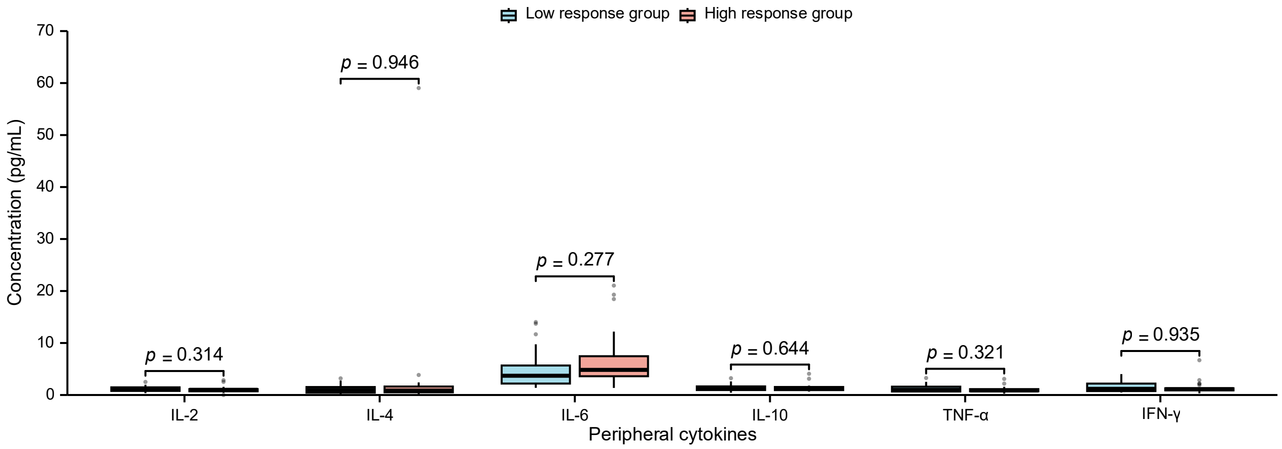

Supplementary Figure 15.

Relationship of tumour response with cytokines in peripheral blood (n = 40). Box plots show median (center line), first and third quartiles (box edges), and 1.5× interquartile range (whiskers). Dots beyond whiskers represent outliers. Statistical analysis was performed using the Mann-Whitney U test (two-sided). $P < 0.05$ was considered statistically significant. Statistical analysis was performed using the Mann-Whitney U test (two-sided). $P < 0.05$ was considered statistically significant. Source data are provided as a Source Data file. IL interleukin, TNF-α tumor necrosis factor-alpha, IFN-γ interferon-gamma.

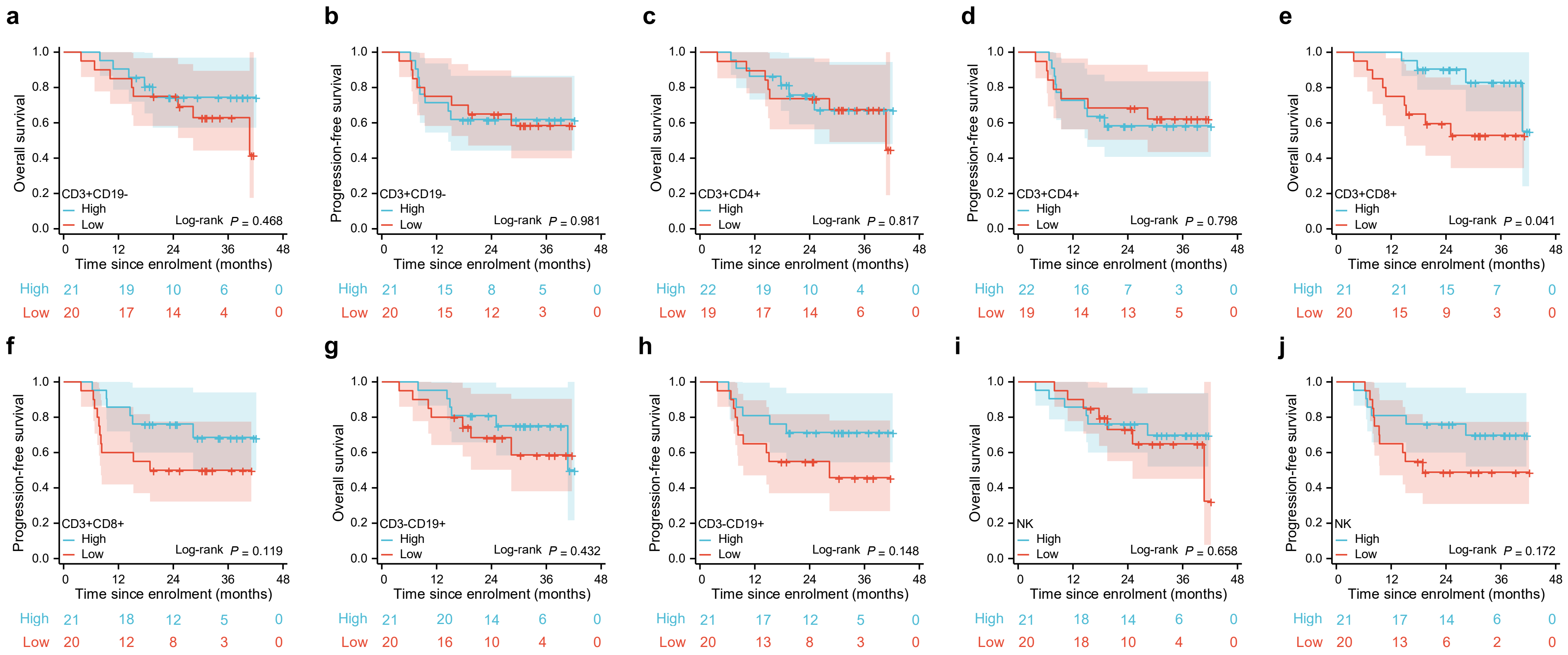

Supplementary Figure 16.

Comparison of treatment outcomes between patients with low and high baseline levels of different lymphocyte subsets in peripheral blood. **(a)** Relationship of overall survival (OS) with CD3+CD19- lymphocytes; **(b)** Relationship of progression-free survival (PFS) with CD3+CD19- lymphocytes; **(c)** Relationship of OS with CD3+CD4+ lymphocytes; **(d)** Relationship of PFS with CD3+CD4+ lymphocytes; **(e)** Relationship of OS with CD3+CD8+ lymphocytes; **(f)** Relationship of PFS with CD3+CD8+ lymphocytes; **(g)** Relationship of OS with CD3-CD19+ lymphocytes; **(h)** Relationship of PFS with CD3-CD19+ lymphocytes; **(i)** Relationship of OS with NK cells; **(j)** Relationship of PFS with NK cells. Kaplan-Meier survival curves are shown with 95% confidence intervals (shaded areas). Statistical analysis was performed using the log-rank test (two-sided). $P < 0.05$ was considered statistically significant. Source data are provided as a Source Data file. NK natural killer.

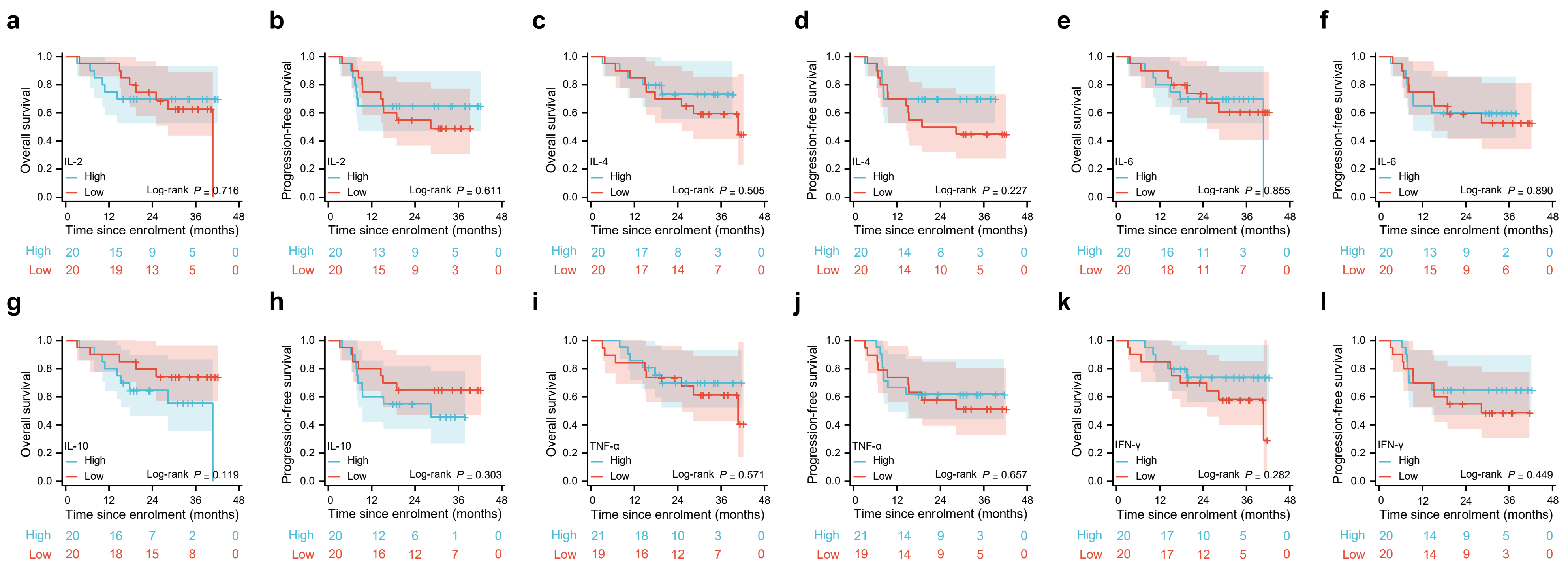

Supplementary Figure 17.

Comparison of treatment outcomes between patients with low and high baseline levels of different cytokines in peripheral blood. **(a)** Relationship of overall survival (OS) with IL-2 levels; **(b)** Relationship of progression-free survival (PFS) with IL-2 levels; **(c)** Relationship of OS with IL-4 levels; **(d)** Relationship of PFS with IL-4 levels; **(e)** Relationship of OS with IL-6 levels; **(f)** Relationship of PFS with IL-6 levels; **(g)** Relationship of OS with IL-10 levels; **(h)** Relationship of PFS with IL-10 levels; **(i)** Relationship of OS with TNF-α levels; **(j)** Relationship of PFS with TNF-α levels; **(k)** Relationship of OS with IFN-γ levels; **(l)** Relationship of PFS with IFN-γ levels. Kaplan-Meier survival curves are shown with 95% confidence intervals (shaded areas). Statistical analysis was performed using the log-rank test (two-sided). $P < 0.05$ was considered statistically significant. Source data are provided as a Source Data file. IL interleukin, TNF-α tumor necrosis factor-alpha, IFN-γ interferon-gamma.

Supplementary Table 1. Treatment compliance

Variable	Value
Induction chemotherapy plus camrelizumab (N=49)	
Completed two planned cycles	49 (100%)
Chemotherapy dose reduction	1 (2.0%)
Reason for chemotherapy dose reduction	
Adverse events (hepatic dysfunction)	1 (2.0%)
Radiotherapy (N=46)	
Completed planned radiotherapy dose	45 (97.8%)
Completed radiotherapy of ≥50 Gy	46 (100%)
Completed radiotherapy of ≥60 Gy	37 (80.4%)
Median duration of radiotherapy (Range)	38 days (31–55)
Reason for premature cessation	
Adverse events (endotoxic shock)	1 (2.2%)
Concurrent chemotherapy (N=46)	
Completed two planned cycles	44 (95.7%)
Dose reduction	6 (13.0%)
Reason for premature cessation	
Poor general conditions	1 (2.2%)
Splenorrhagia	1 (2.2%)
Reason for dose reduction	
Myelosuppression	5 (10.9%)
Hepatic dysfunction	1 (2.2%)

Data are presented as n (%) unless otherwise specified.

Supplementary Table 2. Recurrence pattern (N=49)

Variable	Value
LRR only	5 (10.2%)
Esophagus only	4 (8.2%)
Regional lymph nodes only	1 (2.0%)
Both	0
DM only	4 (8.2%)
Brain	2 (4.1%)
Adrenal gland	1 (2.0%)
Non-regional lymph nodes	1 (2.0%)
LRR and DM	4 (8.2%)
Esophagus and liver	1 (2.0%)
Esophagus, regional lymph nodes and lung	1 (2.0%)
Regional lymph nodes and brain	1 (2.0%)
Regional lymph nodes, brain and adrenal gland	1 (2.0%)

Data are presented as n (%). LRR locoregional recurrence, DM distant metastasis.

Supplementary Table 3. Cause of death (N=49)

PatientsID	Cause of death	Type of progressive disease
1	Grade 5 myelosuppression and septic shock	-
2	Systemic multiple organ failure subsequent to self-administering Chinese medicine	-
3	Unknown	-
4	Unknown	-
5	Infection	-
6	Progressive disease	Distant metastasis in brain
7	Progressive disease	Distant metastasis in adrenal gland
8	Progressive disease	Esophagus recurrence and distant metastasis in liver
9	Progressive disease	Esophagus recurrence
10	Unknown	-
12	Progressive disease	Non-regional lymph nodes recurrence
17	Progressive disease	Esophagus recurrence
22	Progressive disease	Regional lymph nodes recurrence
25	Progressive disease	Esophagus recurrence
28	Progressive disease	Regional lymph nodes recurrence and distant metastasis in brain and adrenal gland
29	COVID-19 infection	-
46	Progressive disease	Regional lymph nodes recurrence and distant metastasis in brain

Supplementary Table 4. Comparison of the patient characteristics in trial and control group before and after propensity score matching

Variable	Before propensity score matching			After propensity score matching		
	Trial group (n = 46)	Control group (n = 71)	*P* value	Trial group (n = 46)	Control group (n = 46)	*P* value
Age (years)			0.181			0.262
< 65	29 (63.0%)	53 (74.6%)		29 (63.0%)	34 (73.9%)	
≥ 65	17 (37.0%)	18 (25.4%)		17 (37.0%)	12 (26.1%)	
Sex			0.532			0.765
Male	39 (84.8%)	63 (88.7%)		39 (84.8%)	40 (87.0%)	
Female	7 (15.2%)	8 (11.3%)		7 (15.2%)	6 (13.0%)	
ECOG performance status			0.090			0.393
0	30 (65.2%)	35 (49.3%)		30 (65.2%)	26 (56.5%)	
1	16 (34.8%)	36 (50.7%)		16 (34.8%)	20 (43.5%)	
Tumor location			0.066			0.400
Middle/Lower	24 (52.2%)	49 (69.0%)		24 (52.2%)	28 (60.9%)	
Upper	22 (47.8%)	22 (31.0%)		22 (47.8%)	18 (39.1%)	
Primary tumor length			0.160			0.342
< 5	10 (21.7%)	24 (33.8%)		10 (21.7%)	14 (30.4%)	
≥ 5	36 (78.3%)	47 (66.2%)		36 (78.3%)	32 (69.6%)	
Clinical T stage			0.773			1.000
T1–2	6 (13.0%)	8 (11.3%)		6 (13.0%)	6 (13.0%)	
T3–4	40 (87.0%)	63 (88.7%)		40 (87.0%)	40 (87.0%)	
Clinical N stage			0.102			0.283
N0–1	15 (32.6%)	34 (47.9%)		15 (32.6%)	20 (43.5%)	
N2–3	31 (67.4%)	37 (52.1%)		31 (67.4%)	26 (56.5%)	
Supraclavicular lymph node involvement			0.165			0.241
No	31 (67.4%)	56 (78.9%)		31 (67.4%)	36 (78.3%)	
Yes	15 (32.6%)	15 (21.1%)		15 (32.6%)	10 (21.7%)	
Clinical TNM stage			0.832			1.000
I–III	21 (45.7%)	31 (43.7%)		21 (45.7%)	21 (45.7%)	
IV	25 (54.3%)	40 (56.3%)		25 (54.3%)	25 (54.3%)	

Data are presented as n (%) unless otherwise specified. Statistical comparisons were performed using chi-square test (two-sided). $P < 0.05$ was considered statistically significant. ECOG Eastern Cooperative Oncology Group.

Supplementary Note

Clinical Research Protocol

A phase 2 trial of induction chemotherapy plus camrelizumab (SHR-1210) followed by concurrent chemoradiotherapy in patients with unresectable locally advanced esophageal squamous cell carcinoma (ImpactCRT)

Clinical Research Protocol

Principal Investigator

Professor Yong Bao
Department of Radiation Oncology, The First Affiliated Hospital of Sun Yat-Sen University, 58 Zhongshan Road II, Guangzhou, 510080, Guangdong Province, China
E-mail: baoyong@mail.sysu.edu.cn

Professor Chao Cheng
Department of Thoracic Surgery, The First Affiliated Hospital of Sun Yat-Sen University, 58 Zhongshan Road II, Guangzhou, 510080, Guangdong Province, China
E-mail: chengch3@mail.sysu.edu.cn

Participating center

The First Affiliated Hospital of Sun Yat-Sen University

Version 2.0
Date：2020.06.11
Approved by the Ethics Committee of the Guangdong Association Study of Thoracic Oncology

Investigator Signatory

I agree to conduct this clinical study in accordance with the design outlined in this protocol and to abide by Chinese laws, the Helsinki Declaration, and Chinese GCP.

Principal Investigator's Signature:
Date:

Contents

1. Summary 5
2. Background 11
3. Objective and Endpoints 12
   3.1 Objective 12
   3.2 Endpoints 12
4. Study Design 13
   4.1 Screening Period 13
   4.2 Induction Therapy Period 13
   4.3 Concurrent Chemoradiotherapy Period 13
   4.4 Follow-up Period 13
5. Study Population 17
   5.1 Inclusion Criteria 17
   5.2 Exclusion Criteria 17
   5.3 Withdrawal Criteria 19
6. Study Treatment 20
   6.1 Induction Phase 20
   6.2 Concurrent Chemoradiotherapy Phase 20
7. Concomitant Medication 22
8. Interruption and Dose Modifications 23
   8.1 Radiotherapy Interruption 23
   8.2 Chemotherapy Suspension 23
   8.3 Chemotherapy Dose Modifications 23
   8.4 Camrelizumab Suspension 23
9. Evaluation 26
   9.1 Efficacy Evaluation 26
   9.2 Safety Evaluation 27
   9.3 Health-related Quality of Life Evaluation 27
   9.4 Exploratory Evaluation 29
10. Statistical Methods 30
11. Adverse Events 31
   11.1 Definition of Adverse Events 31
   11.2 Criteria for the Severity of Adverse Events 31
   11.3 Recording of Adverse Events 31
   11.4 Management of Adverse Events 31
   11.5 Management of Serious Adverse Events 32
   11.6 Management of Tumor Recurrence and Metastasis 32
12. Others 33
   12.1 Informed Consent 33
   12.2 Case Report 33
   12.3 Ethical Requirements 33
   12.4 Quality Control 33
   12.5 Training of Researchers 33

12.6 Improvement of Patients' Compliance 33
12.7 Management of Data 33
12.8 Study Significance 34
13. References 35

1. Summary

**Title**	A phase 2 trial of induction chemotherapy plus camrelizumab (SHR-1210) followed by concurrent chemoradiotherapy in patients with unresectable locally advanced esophageal squamous cell carcinoma.
**Design**	A prospective single-arm phase 2 study
**Objective**	To evaluate the efficacy, safety, and potential biomarkers of induction chemotherapy plus camrelizumab (SHR-1210) followed by concurrent chemoradiotherapy in patients with unresectable locally advanced esophageal squamous cell carcinoma (ESCC), and to provide a reliable theoretical basis for optimizing the comprehensive treatment mode of ESCC.
**Endpoints**	1) Primary endpoint: To evaluate the 1-year survival rate of patients with unresectable locally advanced ESCC treated with camrelizumab combined with induction chemotherapy followed by concurrent chemoradiotherapy;<br>2) Secondary endpoint: To evaluate overall survival (OS), progression-free survival (PFS), objective response rate (ORR), disease control rate (DCR), duration of response (DoR), safety, and health-related quality of life (EORTC QLQ-C30, EORTC QLQ-OES18);<br>3) Exploratory endpoint: To investigate the potential association between tumor tissue and/or blood biomarkers and treatment efficacy.
**Population**	Unresectable locally advanced esophageal squamous cell carcinoma
**Inclusion Criteria**	1) Aged between 18–75 years;<br>2) Histologically confirmed confirmed ESCC;<br>3) No prior treatment;<br>4) Staged cT1–4bN0–3M0 (not suitable for surgery, including inoperability, surgical contraindication, or refusal of surgery) or M1 disease confined to supraclavicular lymph node metastases according to the 8th TNM staging system of the American Joint Committee on Cancer (AJCC);<br>5) Presence of at least one evaluable lesion according to Response Evaluation Criteria in Solid Tumors (RECIST), version 1.1;<br>6) Eastern Cooperative Oncology Group (ECOG) performance status of 0–1;<br>7) Estimated life expectancy of at least 12 weeks;<br>8) Adequate hematologic, cardiac, pulmonary, hepatic, and renal function, as defined below:<br>(1) Hematologic function: Absolute neutrophil count (ANC) $\geq 1.5\times10^9/L$; Platelet count (PLT) $\geq 100\times10^9/L$; Hemoglobin content (HGB) ≥9.0 g/dL;<br>(2) Cardiac function: Myocardial enzymes within the normal range;<br>(3) Pulmonary function: Forced Expiratory Volume in 1 second (FEV1) >0.8 L;

	(4) Hepatic function: Serum total bilirubin (TBIL) ≤1.5×Upper Limit of Normal (ULN); Alanine aminotransferase (ALT) and aspartate aminotransferase (AST) ≤2.5×ULN;<br>(5) Renal function: Serum creatinine ≤1.5×ULN or Creatinine clearance (Ccr) ≥60 mL/min.
**Exclusion Criteria**	1) Multiple primary esophageal cancers;<br>2) History of other primary malignancies, excluding:<br>(1) Complete remission (CR) of malignant tumors for at least 2 years before enrollment and no other treatment was required during the study;<br>(2) Non-melanoma skin cancer or lentigo maligna (LM) that has been adequately treated and has no evidence of disease recurrence;<br>(3) Adequately treated carcinoma in situ (CIS) with no evidence of disease recurrence;<br>3) Esophageal perforation and/or tumor active massive hemorrhage within 2 months prior to enrollment;<br>4) History of thoracic radiation therapy;<br>5) Patients taking other investigational drugs simultaneously;<br>6) Any prior treatment with anti-PD-1, anti-PD-L1, anti-PD-L2, anti-CD137, anti-CTLA-4 antibodies, or any other antibody or drug specifically targeting T-cell costimulation or checkpoint pathways before the study;<br>7) History of allergic reactions attributed to any monoclonal antibody or chemotherapy drugs (paclitaxel, carboplatin) preparations or excipients;<br>8) Patients taking rifampicin, phenytoin sodium, carbamazepine, or barbiturates (these drugs induce CYP3A and may reduce plasma levels of paclitaxel);<br>9) Received systemic therapy of Chinese herbal medicine with anti-tumor indications or immunomodulatory drugs (including thymosin, interferon, interleukin, etc.) within 2 weeks prior to the first administration;<br>10) Administration of a live, attenuated vaccine within 4 weeks prior to the first dose of treatment or planned during the study (administration of inactivated virus vaccine for seasonal influenza is permitted within 4 weeks prior to the first dose of treatment, while live attenuated flu vaccines are not allowed);<br>11) Major surgical procedure within 4 weeks prior to the first dose of treatment or planned during the study;<br>12) Known autoimmune disease that needs symptomatic treatment or history of disease within 2 years (patients with vitiligo, psoriasis, hair loss, or Graves' disease that doesn't need systemic treatment, hypothyroidism that only needs thyroid hormone replacement therapy, and type 1 diabetes which only need insulin replacement therapy can be enrolled);<br>13) Known history of primary immunodeficiency;<br>14) HIV infection and carriers are known to exist (HIV antibody positive);

	15) Known history of allogeneic organ transplantation and allogeneic hematopoietic stem cell transplantation;<br>16) Known active tuberculosis infection;<br>17) Severe infections in the active phase or clinically poorly controlled;<br>18) Uncontrollable clinical cardiac symptoms or diseases, including:<br>(1) Heart failure above NYHA II;<br>(2) Unstable angina pectoris;<br>(3) Myocardial infarction within 1 year;<br>(4) Clinically significant supraventricular or ventricular arrhythmia requiring clinical intervention;<br>19) History of deep vein thrombosis, pulmonary embolism, or any other severe thromboembolism within 3 months prior to enrollment (Implantable Venous Access Port or duct-derived thrombosis, or superficial venous thrombosis is not considered as "severe" thromboembolism);<br>20) Uncontrolled metabolic disorders or other non-malignant organ or systemic diseases or secondary reactions to cancer that may result in higher medical risk and/or uncertainty in the assessment of survival;<br>21) Hepatic encephalopathy, hepatorenal syndrome, and Child-Pugh B liver cirrhosis or worse;<br>22) Active hepatitis B (HBV DNA ≥2000 IU/mL or 10^4 copies/mL), hepatitis C (positive for hepatitis C antibody, and HCV-RNA levels higher than the lower limit of assay);<br>23) Interstitial lung disease (ILD) requiring a steroid therapy;<br>24) Pregnant or lactating female patients;<br>25) Female patients of reproductive age, as well as male patients whose sexual partners are females of reproductive age, refuse to use effective contraceptive measures throughout the treatment period and for six months after the treatment ends；<br>26) Other acute or chronic diseases, psychiatric disorders, or abnormal laboratory test values that may: increase the related risk of study participation or drug administration, interfere with the interpretation of study results, or render patients ineligible for participation at the researcher's discretion;<br>27) Refusal to sign the written informed consent and inability to comply with the visits and related procedures specified in the program.
**Withdraw Criteria**	1) The patient is found to be ineligible for the inclusion/exclusion criteria and is deemed unsuitable for further participation in the study by the investigator;<br>2) The patient violates the study protocol and is deemed unsuitable for further participation in the study by the investigator;<br>3) The patient or their legal representative (e.g., a parent or legal guardian) requests to withdraw from the study or discontinue study drugs (if the patient withdraws informed consent for treatment but not for follow-up, long-term follow-up is still

	available);<br>4) The patient participates in any other type of research that is considered scientifically or medically incompatible with this study;<br>5) The patient needs to be treated with another drug shown to be effective for the study indication and should withdraw from the study before using the new drug;<br>6) The patient develops disease progression, and further treatment is deemed unsuitable by the investigator;<br>7) The patient develops any treatment-related event considered life-threatening;<br>8) The patient develops any other malignancy that requires treatment;<br>9) The patient fails to complete the defined follow-up evaluations (research center staff should contact the patient who has lost follow-up to determine the reason and attempt to reschedule the visit. The date of contact and the contact details should be recorded in the study file);<br>10) The investigator or co-sponsors may terminate the study or discontinue the patient's participation for medical, safety, regulatory, or other reasons related to Good Clinical Practice (GCP).
**Therapeutic Regimen**	**Induction Therapy**<br>In the induction phase, patients will receive two cycles of camrelizumab, albumin-bound paclitaxel, and carboplatin, repeated every 3 weeks, along with premedication to prevent allergic reactions and significant nausea or vomiting as indicated. Details of the induction therapy are as follows:<br>1) Camrelizumab: 200 mg, IV infusion, on day 1 every 21 days;<br>2) Albumin-bound paclitaxel: 260 mg/m^2, IV infusion, on day 1 every 21 days;<br>3) Carboplatin: area under the curve (AUC) of 5, 5 mg/mL/min, IV infusion, on day 1 every 21 days.<br><br>**Concurrent Chemotherapy**<br>In the concurrent chemoradiotherapy phase, patients will receive two cycles of cisplatin and fluorouracil, repeated every 4 weeks, along with premedication to prevent allergic reactions and significant nausea or vomiting as indicated. Details of the concurrent chemotherapy are as follows:<br>1) Cisplatin: 75 mg/m^2, IV infusion, on day 1 every 28 days;<br>2) Fluorouracil: 750 mg/m^2/24 hours for 5 days, IV pump infusion, from day 1 every 28 days.<br><br>**Definitive Radiotherapy**<br>In the concurrent chemoradiotherapy phase, definitive radiotherapy will be delivered with a linear accelerator. Details are as follows:<br>1) Radiation source: X-rays with energy levels of at least 6 MV;<br>2) Radiotherapy technique: Simultaneous integrated boost

	intensity-modulated radiotherapy (SIB-IMRT);<br>3) Patient positioning: All patients will be positioned supine and immobilized using a vacuum bag or body mold;<br>4) Target volume:<br>(1) Gross tumor volume (GTV) encompasses primary esophageal tumor (GTVp) and positive lymph nodes (GTVn);<br>(2) Clinical target volume (CTV) encompasses GTVp along with a superior and inferior expansion of 3 cm along the length of the esophagus, as well as a radial expansion of 0.5–1.0 cm, and the GTVn plus a margin of 0.5–1.0 cm, including coverage of elective nodal regions. Elective treatment of node-bearing regions depends on the location of the primary tumor in the esophagus and Esophagogastric Junction Cancers (EGJ): a. Cervical esophagus: Consider treatment of the supraclavicular nodes and treatment of higher echelon cervical nodes, especially if the nodal stage is N1 or greater; b. Proximal third of the esophagus: Consider treatment of para-esophageal lymph nodes and supraclavicular lymph nodes; c. Middle third of the esophagus: Consider treatment of para-esophageal lymph nodes; d. Distal third of esophagus and EGJ: Consider para-esophageal, lesser curvature, and celiac axis nodal regions;<br>(3) Planning gross tumor volume (PGTV) is expanded by 0.6–0.8 cm based on GTV;<br>(4) Planning target volume (PTV) is expanded by 0.6–0.8 cm based on CTV;<br>5) Prescribed dose: The total doses of the PTV and PGTV are 50–50.4 Gy and 50–63 Gy, respectively, both delivered in 25–28 fractions, five times per week;<br>6) Organs at risk (OARs):<br>(1) Lung: Dmean <17 Gy, V30 <20%, V20 <30%, V5 <65%;<br>(2) Heart: Dmean ≤26 Gy, V30 <40%;<br>(3) Spinal cord: Dmax ≤45 Gy.
**Sample Size**	A total sample size of 44 patients are necessary to warrant a power of 80% at a one-sided α level of 0.05 to demonstrate an improvement of 14% in the 1-year survival rate (from 66% in the previous study [RTOG 9405] to 80% in the current study), assuming an accrual period of 18 months and a minimum follow-up period of 12 months. Accounting for a potential dropout rate of up to 10%, the final estimated sample size is 49 patients.
**Statistical Methods**	The Kaplan-Meier method will be employed to estimate OS and PFS along with their corresponding 95% confidence intervals (CIs). A log-rank test will be performed to assess survival differences between groups, and Cox proportional hazards regression will be used to estimate hazard ratios (HRs) and 95% CIs. The analysis of efficacy and safety will encompass all patients enrolled in the intention-to-treat (ITT) population during induction therapy and the per-protocol population throughout the entire treatment duration. All reported P values are two-sided with significance levels set at 0.05.

**Version Number**	2.0

2. Background

Esophageal cancer (EC) is one of the most common malignancies globally, ranking seventh in terms of incidence and sixth in mortality overall worldwide in 2018[1]. More than 50% of cases occurred in China[2]. Esophageal squamous cell carcinoma (ESCC) is the most common type, particularly in high-risk regions, where it represents more than 90% of all EC patients[3-5]. With no specific symptoms of early EC, most patients are diagnosed at a late stage and rapidly progress to an advanced stage, when treatment options are limited. Definitive chemoradiotherapy (CRT) can significantly improve local control and reduce distant metastasis, thereby prolonging patient survival, and has become the standard of care for patients with locally advanced esophageal cancer. However, the treatment efficacy remains unsatisfactory, with a 3-year overall survival rate ranging from 27% to 33%. Furthermore, the treatment regimens have not changed since the 1990s[6-9]. Therefore, more effective strategies are urgently needed for this patient population.

Immunotherapy, particularly Programmed cell death-1 (PD-1)/Programmed cell death-ligand 1 (PD-L1) inhibitor therapy, has substantially improved the prognosis of various tumors[10]. PD-1, an immunoinhibitory receptor, is mainly expressed on the membrane surface of activated T lymphocytes, B lymphocytes, NK cells, dendritic cells, and monocytes[11]. PD-L1, the main ligand of PD-1, can be expressed in antigen-presenting cells, vascular endothelial cells, testis, placenta, cornea, and other cells[12]. Binding of PD-L1 with PD-1 can significantly inhibit the function of cytotoxic T cells, induce the production of regulatory T cells, and regulate the secretion and expression of cytokines[12,13]. High expression of PD-1 and PD-L1 in a variety of malignant tumor tissues contributes to tumor cell immune escape and poor prognosis, including EC[14-17]. Therefore, immune checkpoint inhibitors targeting the PD-1/PD-L1 pathway may provide a promising therapeutic option for patients with EC.

Immunotherapy combined with chemotherapy and/or radiotherapy has been widely established to play a synergistic anti-tumor effect[18-23]. Currently, advanced EC has entered the era of immunotherapy, particularly PD-1 inhibitor therapy, which has shown good efficacy and safety. Anti-PD-1 antibodies, when used as monotherapy, have shown promising activity and a manageable safety profile in the second-line or further-line settings for advanced EC[24-29]. Moreover, several phase III clinical trials are underway to investigate the efficacy and safety of combining PD-1 inhibitors with chemotherapy as a first-line treatment for advanced EC, including CheckMate-648, ESCORT-1st, KEYNOTE-590, ORIENT-15, JUPITER-06, RATIONALE-306, ASTRUM-007, and others. Notably, clinical trials on immunotherapy combined with chemoradiotherapy for locally advanced EC are sparse, and no data have been reported to date.

In this context, this study intends to conduct a prospective, single-arm, phase II clinical study on the activity and safety of induction chemotherapy plus camrelizumab followed by concurrent chemoradiotherapy for patients with unresectable locally advanced ESCC.

3. Objective and Endpoints

3.1 Objective

To evaluate the efficacy, safety, and potential biomarkers of induction chemotherapy plus camrelizumab (SHR-1210) followed by concurrent chemoradiotherapy in patients with unresectable locally advanced ESCC, and to provide a reliable theoretical basis for optimizing the comprehensive treatment mode of ESCC.

3.2 Endpoints

The endpoints of the study are as follows:

1) Primary endpoint: To evaluate the 1-year survival rate of patients with unresectable locally advanced ESCC treated with camrelizumab combined with induction chemotherapy followed by concurrent chemoradiotherapy;

2) Secondary endpoint: To evaluate overall survival (OS), progression-free survival (PFS), objective response rate (ORR), disease control rate (DCR), duration of response (DoR), safety, and health-related quality of life (EORTC QLQ-C30, EORTC QLQ-OES18);

3) Exploratory endpoint: To investigate the potential association between tumor tissue and/or blood biomarkers and treatment efficacy.

4. Study Design

The study design schematic is presented in Figure 1 and Table 1.

4.1 Screening Period

Patients will be fully informed about the nature of the study before conducting research related tests. Screening evaluations will be performed within 28 days prior to the first dose administration. The investigator will assess patient eligibility according to the screening assessment results. Patients who meet the criteria and agree to sign informed consent forms will be recruited into the study.

4.2 Induction Therapy Period

After completing all screening activities, patients confirmed to be eligible will receive two cycles of camrelizumab (200 mg) combined with albumin-bound paclitaxel (260 mg/m^2) and carboplatin (area under the curve of 5), repeated every 3 weeks.

During this period, the safety of the treatment and health-related quality of life will be evaluated. After completing two cycles of induction treatment and before starting concurrent chemoradiotherapy, the patient must return to the hospital for re-examination to evaluate efficacy and exclude any contraindications for radiotherapy.

4.3 Concurrent Chemoradiotherapy Period

Within 3–4 weeks after completing induction treatment, the patient will receive concurrent chemoradiotherapy. Definitive radiotherapy will be delivered with a linear accelerator of at least 6 MV using simultaneous integrated boost intensity-modulated radiotherapy (SIB-IMRT). During radiotherapy, two cycles of Cisplatin (75 mg/m^2) and Fluorouracil (750 mg/m^2/24 hours for 5 days) will be administered on the first day of each cycle, once every four weeks.

During this period, the safety of the treatment will be evaluated. One month after completing concurrent chemoradiotherapy, the patient must return to the hospital for re-examinationt to evaluate efficacy.

4.4 Follow-up Period

Subsequent follow-up evaluations will be conducted every 3 months until disease progression. Investigators will review all examination results and make a disease assessment.

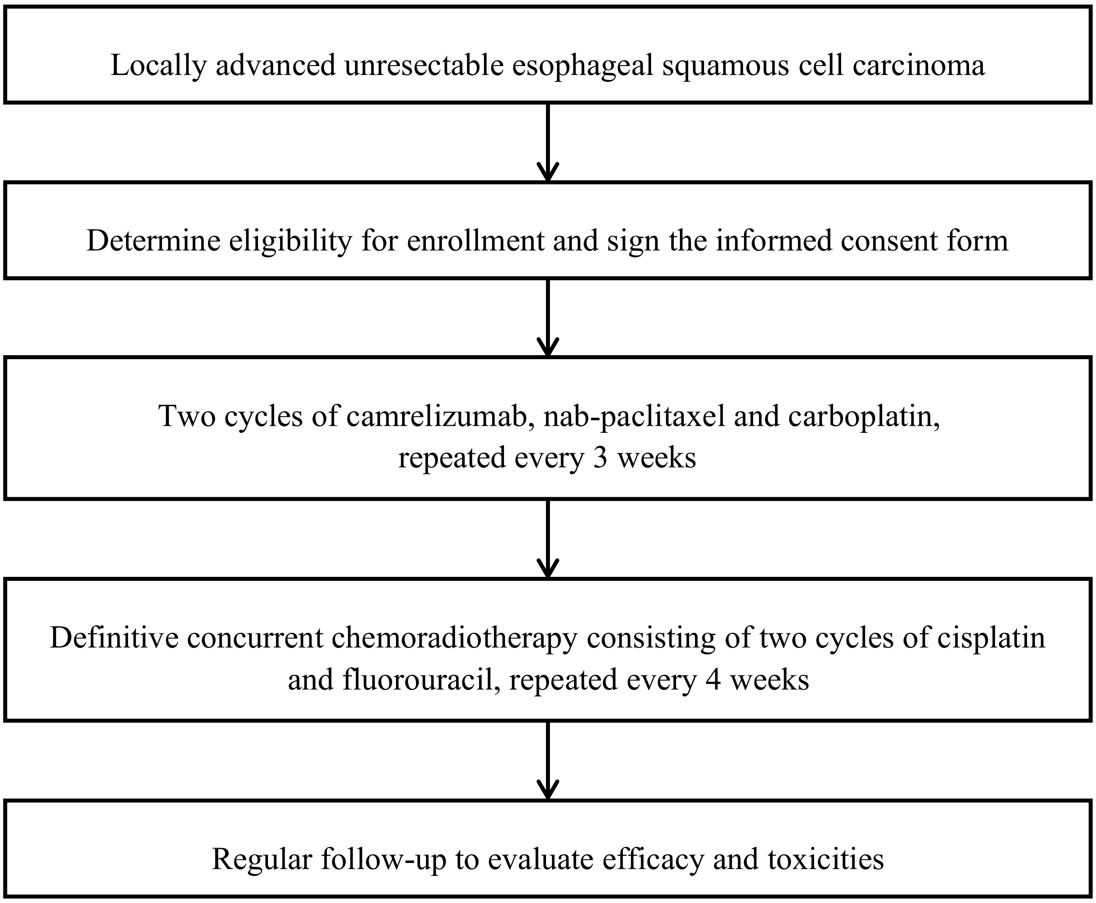

Figure 1. Study design

Table 1. Study schedule

Items / Period	Screening	Induction therapy		Concurrent chemoradiotherapy			Evaluation	Follow-up
	≤-4w	First cycle	Second cycle	Before	During	After	1 month (±3 days)	Once per 3 months
General study process								
Written informed consent	√							
Verify eligibility criteria	√							
Demographic characteristic	√							
Medical history	√							
Vital signs	√	√	√	√	√	√	√	
ECOG performance status score	√	√	√	√	√	√	√	
Physical examination	√	√	√	√	√	√	√	
Laboratory tests								
Complete blood count	√	√	√	√	√	√	√	
Urinalysis	√			√				
Routine stool test	√			√				
Blood biochemistry tests	√	√	√	√	√	√	√	
Coagulation test	√	√	√	√	√	√	√	
Myocardial enzyme profile	√	√	√	√	√	√	√	
Thyroid function test	√			√		√	√	
Blood cortisol	√			√		√	√	
Potential infection screening tests	√					√		
Digestive system tumor markers	√	√	√	√		√	√	
β-HCG test	√							
Auxiliary examinations								
Electrocardiogram	√	√	√	√	√	√		

Echocardiography	√							
Pulmonary function test	√							
PET-CT or enhanced CT of neck, chest and upper abdomen	√			√	√	√	√	√
Esophageal MRI	√			√		√	√	√
ECT	√							
Barium esophagogram	√			√				
Esophagogastroscopy with endoscopic ultrasound	√			√				
Bronchoscopy	√ [a]							
Safety and efficacy evaluation								
Adverse events evaluation		√	√	√	√	√	√	
Efficacy evaluation				√			√	
Quality of life evaluation								
QLQ-C30	√			√				
QLQ-OES18	√			√				
Specimen Collection								
Peripheral blood	√			√				
Biopsy tissue	√							
Survival Outcome								
Time of disease progression	√						√	√
Time of death	√						√	√

[a] A bronchoscopy will be required when the tumour was suspected of invading the trachea or bronchus on CT or endoscopic ultrasound.

QLQ-C30 Quality of Life Questionnaire-Core 30, *QLQ-OES18* Quality of Life Questionnaire-Esophageal Cancer Module-18.

5. Study Population

5.1 Inclusion Criteria

The inclusion criteria for patients are as follows:

1) Aged between 18–75 years;

2) Histologically confirmed confirmed ESCC;

3) No prior treatment;

4) Staged cT1–4bN0–3M0 (not suitable for surgery, including inoperability, surgical contraindication, or refusal of surgery) or M1 disease confined to supraclavicular lymph node metastases according to the 8th TNM staging system of the American Joint Committee on Cancer (AJCC);

5) Presence of at least one evaluable lesion according to Response Evaluation Criteria in Solid Tumors (RECIST), version 1.1;

6) Eastern Cooperative Oncology Group (ECOG) performance status of 0–1;

7) Estimated life expectancy of at least 12 weeks;

8) Adequate hematologic, cardiac, pulmonary, hepatic, and renal function, as defined below:

(1) Hematologic function: Absolute neutrophil count (ANC) $\geq 1.5\times10^9$/L; Platelet count (PLT) $\geq 100\times10^9$/L; Hemoglobin content (HGB) ≥ 9.0 g/dL;

(2) Cardiac function: Myocardial enzymes within the normal range;

(3) Pulmonary function: Forced Expiratory Volume in 1 second (FEV1) >0.8 L;

(4) Hepatic function: Serum total bilirubin (TBIL) $\leq 1.5\times$Upper Limit of Normal (ULN); Alanine aminotransferase (ALT) and aspartate aminotransferase (AST) $\leq 2.5\times$ULN;

(5) Renal function: Serum creatinine $\leq 1.5\times$ULN or Creatinine clearance (Ccr) ≥ 60 mL/min.

5.2 Exclusion Criteria

The exclusion criteria for patients are as follows:

1) Multiple primary esophageal cancers;

2) History of other primary malignancies, excluding:

(1) Complete remission (CR) of malignant tumors for at least 2 years before enrollment and no other treatment was required during the study;

(2) Non-melanoma skin cancer or lentigo maligna (LM) that has been adequately treated and has no evidence of disease recurrence;

(3) Adequately treated carcinoma in situ (CIS) with no evidence of disease recurrence;

3) Esophageal perforation and/or tumor active massive hemorrhage within 2 months prior to enrollment;

4) History of thoracic radiation therapy;

5) Patients taking other investigational drugs simultaneously;

6) Any prior treatment with anti-PD-1, anti-PD-L1, anti-PD-L2, anti-CD137, anti-CTLA-4 antibodies, or any other antibody or drug specifically targeting T-cell costimulation or checkpoint pathways before the study;

7) History of allergic reactions attributed to any monoclonal antibody or chemotherapy drugs (paclitaxel, carboplatin) preparations or excipients;

8) Patients taking rifampicin, phenytoin sodium, carbamazepine, or barbiturates (these drugs induce CYP3A and may reduce plasma levels of paclitaxel);

9) Received systemic therapy of Chinese herbal medicine with anti-tumor indications or immunomodulatory drugs (including thymosin, interferon, interleukin, etc.) within 2 weeks prior to the first administration;

10) Administration of a live, attenuated vaccine within 4 weeks prior to the first dose of treatment or planned during the study (administration of inactivated virus vaccine for seasonal influenza is permitted within 4 weeks prior to the first dose of treatment, while live attenuated flu vaccines are not allowed);

11) Major surgical procedure within 4 weeks prior to the first dose of treatment or planned during the study;

12) Known autoimmune disease that needs symptomatic treatment or history of disease within 2 years (patients with vitiligo, psoriasis, hair loss, or Graves' disease that doesn't need systemic treatment, hypothyroidism that only needs thyroid hormone replacement therapy, and type 1 diabetes which only need insulin replacement therapy can be enrolled);

13) Known history of primary immunodeficiency;

14) HIV infection and carriers are known to exist (HIV antibody positive);

15) Known history of allogeneic organ transplantation and allogeneic hematopoietic stem cell transplantation;

16) Known active tuberculosis infection;

17) Severe infections in the active phase or clinically poorly controlled;

18) Uncontrollable clinical cardiac symptoms or diseases, including:

(1) Heart failure above NYHA II;

(2) Unstable angina pectoris;

(3) Myocardial infarction within 1 year;

(4) Clinically significant supraventricular or ventricular arrhythmia requiring clinical intervention;

19) History of deep vein thrombosis, pulmonary embolism, or any other severe thromboembolism within 3 months prior to enrollment (Implantable Venous Access Port or duct-derived thrombosis, or superficial venous thrombosis is not considered as "severe" thromboembolism);

20) Uncontrolled metabolic disorders or other non-malignant organ or systemic diseases or secondary reactions to cancer that may result in higher medical risk and/or uncertainty in the assessment of survival;

21) Hepatic encephalopathy, hepatorenal syndrome, and Child-Pugh B liver cirrhosis or worse;

22) Active hepatitis B (HBV DNA ≥2000 IU/mL or 10^4 copies/mL), hepatitis C (positive for hepatitis C antibody, and HCV-RNA levels higher than the lower limit of assay);

23) Interstitial lung disease (ILD) requiring a steroid therapy;

24) Pregnant or lactating female patients;

25) Female patients of reproductive age, as well as male patients whose sexual partners are females of reproductive age, refuse to use effective contraceptive measures

throughout the treatment period and for six months after the treatment ends；

26) Other acute or chronic diseases, psychiatric disorders, or abnormal laboratory test values that may: increase the related risk of study participation or drug administration, interfere with the interpretation of study results, or render patients ineligible for participation at the researcher's discretion;

27) Refusal to sign the written informed consent and inability to comply with the visits and related procedures specified in the program.

5.3 Withdrawal Criteria

The withdrawal criteria for patients are as follows:

1) The patient is found to be ineligible for the inclusion/exclusion criteria and is deemed unsuitable for further participation in the study by the investigator;

2) The patient violates the study protocol and is deemed unsuitable for further participation in the study by the investigator;

3) The patient or their legal representative (e.g., a parent or legal guardian) requests to withdraw from the study or discontinue study drugs (if the patient withdraws informed consent for treatment but not for follow-up, long-term follow-up is still available);

4) The patient participates in any other type of research that is considered scientifically or medically incompatible with this study;

5) The patient needs to be treated with another drug shown to be effective for the study indication and should withdraw from the study before using the new drug;

6) The patient develops disease progression, and further treatment is deemed unsuitable by the investigator;

7) The patient develops any treatment-related event considered life-threatening;

8) The patient develops any other malignancy that requires treatment;

9) The patient fails to complete the defined follow-up evaluations (research center staff should contact the patient who has lost follow-up to determine the reason and attempt to reschedule the visit. The date of contact and the contact details should be recorded in the study file);

10) The investigator or co-sponsors may terminate the study or discontinue the patient's participation for medical, safety, regulatory, or other reasons related to Good Clinical Practice (GCP).

6. Study Treatment

6.1 Induction Phase

In the induction phase, patients will receive two cycles of camrelizumab, albumin-bound paclitaxel, and carboplatin, repeated every 3 weeks, along with premedication to prevent allergic reactions and significant nausea or vomiting as indicated. Details of the induction therapy are as follows:

1) Camrelizumab: 200 mg, IV infusion, on day 1 every 21 days;

2) Albumin-bound paclitaxel: 260 mg/m², IV infusion, on day 1 every 21 days;

3) Carboplatin: area under the curve (AUC) of 5, 5 mg/mL/min, IV infusion, on day 1 every 21 days.

6.2 Concurrent Chemoradiotherapy Phase

Patients will receive concurrent chemoradiotherapy (CCRT) 3–4 weeks after the end of induction therapy.

6.2.1 Concurrent Chemotherapy

In the concurrent chemoradiotherapy phase, patients will receive two cycles of cisplatin and fluorouracil, repeated every 4 weeks, along with premedication to prevent allergic reactions and significant nausea or vomiting as indicated. Details of the concurrent chemotherapy are as follows:

1) Cisplatin: 75 mg/m², IV infusion, on day 1 every 28 days;

2) Fluorouracil: 750 mg/m²/24 hours for 5 days, IV pump infusion, from day 1 every 28 days.

6.2.2 Definitive Radiotherapy

In the concurrent chemoradiotherapy phase, definitive radiotherapy will be delivered with a linear accelerator. Details are as follows:

1) Radiation source: X-rays with energy levels of at least 6 MV;

2) Radiotherapy technique: Simultaneous integrated boost intensity-modulated radiotherapy (SIB-IMRT);

3) Patient positioning: All patients will be positioned supine and immobilized using a vacuum bag or body mold;

4) Target volume:

(1) Gross tumor volume (GTV) encompasses primary esophageal tumor (GTVp) and positive lymph nodes (GTVn);

(2) Clinical target volume (CTV) encompasses GTVp along with a superior and inferior expansion of 3 cm along the length of the esophagus, as well as a radial expansion of 0.5–1.0 cm, and the GTVn plus a margin of 0.5–1.0 cm, including coverage of elective nodal regions. Elective treatment of node-bearing regions depends on the location of the primary tumor in the esophagus and Esophagogastric Junction Cancers (EGJ): a. Cervical esophagus: Consider treatment of the supraclavicular nodes and treatment of higher echelon cervical nodes, especially if the nodal stage is N1 or greater; b. Proximal third of the esophagus: Consider treatment of para-esophageal lymph nodes and supraclavicular lymph nodes; c. Middle third of the esophagus: Consider treatment of para-esophageal lymph nodes; d. Distal third of esophagus and EGJ: Consider para-esophageal, lesser curvature, and celiac axis nodal regions;

(3) Planning gross tumor volume (PGTV) is expanded by 0.6–0.8 cm based on

GTV;

(4) Planning target volume (PTV) is expanded by 0.6–0.8 cm based on CTV;

5) Prescribed dose: The total doses of the PTV and PGTV are 50–50.4 Gy and 50–63 Gy, respectively, both delivered in 25–28 fractions, five times per week;

6) Organs at risk (OARs): Normal organ dose constraints should be taken into consideration as shown in Table 2.

Table 2. Dose restrictions of organs at risk

Organs at risk	Dose constraints
Lung	Dmean (mean dose) <17 Gy, V30 (volume receiving ≥30 Gy) <20%, V20 (volume receiving ≥20 Gy) <30%, V5 (volume receiving ≥5 Gy) <65%
Heart	Dmean (mean dose) ≤26 Gy, V30 (volume receiving ≥30 Gy) <40%
Spinal cord	Dmax (maximum dose) ≤45 Gy
Stomach	Dmean (mean dose) <45 Gy, Dmax (maximum dose) <54 Gy
Bowel	Dmax (maximum dose) <54 Gy, V50 (volume receiving ≥50 Gy) <10%
Liver	V30 (volume receiving ≥30 Gy) ≤20%, V20 (volume receiving ≥20 Gy) ≤30%, Dmean (mean dose) <25 Gy
Kidney	(evaluate each one separately)<br>V20 (volume receiving ≥20 Gy) ≤33%, Dmean (mean dose) <18 Gy

7. Concomitant Medication

The following conditions permit the use of concomitant drugs during the study:

1) When adverse reactions occur in the test, these reactions should be strictly observed and treated. All concomitant drugs should be recorded with explanations on the CRF form;

2) When patients vomit due to chemotherapy, antiemetic agents can be given;

3) Neurotrophic agents, such as adenosine cobalamin and vitamin B, can be used when patients develop neurotoxicity;

4) When the patient has pain that affects quality of life, effective analgesic treatment should be given;

5) When the patient has symptoms such as constipation or diarrhea, symptomatic drugs can be given;

6) If severe myelosuppressive toxicity (Grade 3 or 4 toxicity) occurs during treatment, Granulocyte Colony-Stimulating Factor (G-CSF) and other treatments may be given.

Drugs that cannot be used during treatment are as follows:

1) Other biotherapies (including but not limited to interferon, IL-2, thymosin, immunocell therapy, etc.) and other systemic chemotherapy are prohibited during treatment;

2) Immunotherapy not specified in this protocol is prohibited during treatment;

3) Live vaccine inoculation is prohibited within 28 days before and during drug administration.

8. Interruption and Dose Modifications

8.1 Radiotherapy Interruption

If the following toxicities are observed, radiotherapy must be delayed until they are reduced to grade 1 or lower:

1) WBC < 2.0×10^9/L or ANC < 1.0×10^9/L;

2) PLT < 50×10^9/L;

3) Grade 3 or higher non-hematological toxicity.

If the following toxicity is observed, radiotherapy must be delayed until complete recovery:

1) Thoracic infection with a fever over 38.5°C.

A suspension of up to 2 weeks is allowed; otherwise, radiotherapy will be terminated.

8.2 Chemotherapy Suspension

If the following toxicities are observed, chemotherapy must be delayed until they are reduced to grade 1 or lower:

1) ANC < 1.5×10^9/L;

2) PLT < 100×10^9/L;

3) Grade 2 or higher non-hematological toxicity, except for nausea, vomiting, and alopecia.

8.3 Chemotherapy Dose Modifications

Chemotherapy dose modifications will be based on the highest toxicity observed during the previous cycle. Patients requiring dose modifications will receive the adjusted dose in subsequent cycles. If modifications are necessary, the dose will be reduced by 25% from the planned dose for the first time, and by 50% for the second time. Dose modifications are allowed at most twice; otherwise, chemotherapy will be terminated. Details are as follows:

1) Grade 4 neutropenia;

2) Grade 3 or higher thrombocytopenia;

3) Grade 2 or higher non-hematological adverse events (except for nausea, vomiting, and alopecia).

8.4 Camrelizumab Suspension

Adverse events related to camrelizumab may be immunologically related (irAEs), which may occur shortly after the first administration or several months after the last administration. No dose reduction will be allowed for camrelizumab. In case of the conditions listed in Table 3, the use of camrelizumab should be suspended according to protocol.

Table 3. Immune-related adverse events and management

Adverse event	Grade	Management
Pneumonia	Grade 2	Discontinue for short [a]
	Grade 3 or 4	Discontinue permanently
Diarrhea/enterocolitis	Grade 2 or 3	Discontinue

		for short [a]
	Grade 4	Discontinue permanently
Dermatitis	Grade 3	Discontinue for short [a]
	Grade 4	Discontinue permanently
Hepatitis	For patients with normal baseline ALT, AST, or TBIL, there appears Grade 2 elevation of AST, ALT, or TBIL. For patients with baseline AST, ALT, or TBIL > ULN, AST, ALT, or TBIL elevates ≥ 50% and maintains < 7 days.	Discontinue for short [a]
	For patients with normal baseline ALT, AST, or TBIL, there appears Grade 3 or 4 elevation of AST, ALT, or TBIL. For patients with baseline AST, ALT, or TBIL > ULN, AST, ALT, or TBIL elevates ≥ 50% and maintains ≥ 7 days	Discontinue permanently
Hypophysitis	Grade 2	Discontinue for short [b]
	Grade 3 or 4	Discontinue permanently
Adrenocortical insufficiency	Grade 2	Discontinue for short [b]
	Grade 3 or 4	Discontinue permanently
Hyperthyroidism	Grade 3 or 4	Discontinue permanently
Type 1 diabetes	Grade 3	Discontinue for short [b]
	Grade 4	Discontinue permanently
Renal insufficiency	Grade 2 or 3/elevation of Cr	Discontinue for short [a]
	Grade 4/elevation of Cr	Discontinue permanently
Neurotoxicity	Grade 2	Discontinue for short [a]
	Grade 3 or 4	Discontinue permanently
Infusion reaction	Grade 3 or 4	Discontinue permanently
Other AE	Other Grade 3 AE appears for the first time	Discontinue for short [a]
	The same grade 3 AE occurred for second time	Discontinue permanently
	Grade 3 AE which cannot fall to level 0-2/baseline within 7 days or recover to level	Discontinue permanently

	0-1/baseline level within 14 days	
	Grade 4 AE	Discontinue permanently [c]

[a] Resuming dosing after symptom improvement to level 0-1 or baseline.

[b] Pituitaritis, adrenocortical insufficiency, and type 1 diabetes mellitus can be re-administered if they are adequately controlled and only physiologic hormone replacement therapy is required.

[c] In the case of abnormal grade 4 laboratory results, the decision to discontinue medication should be based on concomitant clinical symptoms/signs and the investigator's clinical judgment.

9. Evaluation

9.1 Efficacy Evaluation

Tumor response will be evaluated prior to the onset of radiotherapy and reassessed one month after completing chemoradiotherapy, in accordance with the Response Evaluation Criteria in Solid Tumors, version 1.1. Subsequent evaluations will be conducted every 3 months until disease progression. Tumor response to induction therapy was assessed primarily by neck, chest, and upper abdomen enhanced CT, esophageal MRI, and esophagogastroscopy with endoscopic ultrasound, while tumor response after CRT was assessed primarily by CT and MRI. Evaluation criteria are as follows:

1) Complete response (CR) is defined as the disappearance of all target lesions, and all pathological lymph nodes (including target and non-target nodes) must have reduction in short axis to <10 mm. Considering that the esophagus is a hollow organ, the assessment of a clinical complete remission (cCR) of primary esophageal lesions depends not only on CT imaging examinations but also on the use of various methods such as endoscopy with biopsy, endoscopic ultrasound, and positron emission tomography-computed tomography (PET-CT) to confirm the status. In the current study, efficacy was assessed primarily by CT and MRI, warranting additional caution when evaluating CR of esophageal lesions.

2) Partial response (PR) is defined as at least a 30% decrease in the sum of diameters of target lesions, taking as reference the baseline sum diameters.

3) Progressive disease (PD) is defined as at least a 20% increase in the sum of diameters of target lesions (an absolute increase of at least 5 mm), or the appearance of one or more new lesions.

4) Stable disease (SD) is defined as neither sufficient decrease to qualify for partial response nor sufficient increase to qualify for progressive disease.

Note: According to imaging evaluation, the longest diameter of esophageal lesions is defined as the sum of the maximum diameters of the longitudinal axis and the horizontal axis, in which the maximum diameters of the horizontal axis are defined as the length of the maximum cross-section of the tumor minus the length of the central cavity on the same measurement line.

The definitions of overall survival (OS), progression-free survival (PFS), objective response rate (ORR), disease control rate (DCR), and duration of response (DoR) are as follows:

1) Overall survival (OS): defined as the time from the date of first dose administration to the date of death due to any cause;

2) Progression-free survival (PFS): defined as the time from the date of first dose administration to the first documented disease progression or the date of death due to any cause, whichever occurs first;

3) Objective response rate (ORR): defined as the proportion of patients who achieve a complete response (CR) or partial response (PR);

4) Disease control rate (DCR): defined as the proportion of patients who had no evidence of progression from physical or imaging examinations;

5) Duration of response (DoR): defined as the time from the first observation of complete response or partial response to the date of first disease progression.

9.2 Safety Evaluation

Throughout the study, adverse events will be systematically assessed and graded weekly in accordance with the National Cancer Institute Common Terminology Criteria for Adverse Events, Version 5.0 (NCI CTCAE V5.0). The relationship with the study drug will be judged and recorded on the CRF. Adverse events occurring later than 28 days after the end of treatment will only be recorded if they are considered relevant.

9.3 Health-related Quality of Life Evaluation

Health-related quality of life will be assessed according to the European Organization for Research and Treatment of Cancer (EORTC) Quality of Life Questionnaire-Core 30 (QLQ-C30) and EORTC Quality of Life Questionnaire-Esophageal Cancer Module-18 (QLQ-OES18) scales, at the start of study therapy and again after the induction therapy.

The EORTC QLQ-C30 scale comprises 30 items that are combined to form 5 functioning scales (physical, role, cognitive, emotional, and social), 3 symptom scales (fatigue, pain, and nausea or vomiting), a global health status quality-of-life scale, and 6 single-item scales (dyspnea, insomnia, appetite loss, constipation, diarrhea, and financial difficulties).

The EORTC QLQ-OES18 scale contains 18 items for patients with esophageal cancer, forming 10 symptom scales: pain, reflux, dysphagia, dry mouth, choking when swallowing, and trouble with coughing, eating, swallowing saliva, tasting, and talking.

The scoring method for the EORTC QLQ-C30 and QLQ-OES18 is systematically outlined in Tables 4 and 5, respectively.

Table 4. Scoring method for the EORTC QLQ—C30

Scale (dimension)	Code	Property	Number of Items	Item Range (R)
Physical Functioning	PF	Functional scale	5	3
Role Functioning	RF	Functional scale	2	3
Emotional Functioning	EF	Functional scale	4	3
Cognitive Functioning	CF	Functional scale	2	3
Social Functioning	SF	Functional scale	2	3
Global Health Status	QL		2	6
Fatigue	FA	Symptom scale	3	3
Nausea and Vomiting	NV	Symptom scale	2	3
Pain	PA	Symptom scale	2	3
Dyspnoea	DY	Symptom scale	1	3
Insomnia	SL	Symptom scale	1	3
Appetite Loss	AP	Symptom scale	1	3
Constipation	CO	Symptom scale	1	3

Diarrhea	DI	Symptom scale	1	3
Financial Difficulties	FI	Symptom scale	1	3

Note:
1. Item score calculation
EORTC's QLQ-C3O (V3) is a core scale for all cancer patients, including a total of 30 items. Item 29 and 30 has 7-point scales, scoring 1-7 points depending on the answer. Other items have 4-point scales: Not at all, A little, Quite a bit, and Very much, scoring 1-4 points.
2. Calculation of scale scores (raw scores)
For the convenience of statistical analysis and application, the scale is often divided into scales. A scale is an aspect of a quality of life component, also known as a dimension, which is analyzed as an independent variable. The EORTC QLQ—C30 (V3) scale comprises 30 items divided into 15 scales, including 5 functional scales (physical, role, cognitive, emotional and social), 3 symptom scales (fatigue, pain and nausea/vomiting), 1 global health status/quality of life scale, and 6 singleitem scales (each one is a scale). To get the score of each scale, add up the scores of the items in each scale and divide by the number of items in each scale (Raw Score, RS).
3. Calculation of standard scores
To compare the scores between each scale, a linear transformation is further carried out to standardize the RS so that the standard score (SS) ranges from 0-100. In addition, another purpose of the transformation is to reverse the direction of the score. Except for item 29 and 30 which are reversed items (the larger the score, the worse the quality of life), the scoring rules for QLQ-C30 clearly state that the higher the score for functional scale and global health status, the higher level of function status and QoL, but a high score for symptom status represents a high level of symptoms/problems (worse QoL). Therefore, the score of functional scale needs to be reversed when being standardized. Specifically, the following formula is used (where R is item range).
Functional scale: $SS = [1 - (RS - 1)/R] \times 100$
Global health status, symptom scale and 6 single-item scales: $SS = [(RS - 1)/R] \times 100$
Handling of missing data: For the global health status, functional scale, and symptom scale, if the number of answered items in the scale reaches ≥ 50%, the score is calculated using the above steps; otherwise the score of this scale is considered missing.

Table 5. Scoring method for the EORTC QLQ—OES18

Scale (dimension)	Code	Number of Items	Item Range (R)
Dysphagia	OESDYS	3	3
Eating	OESEAT	4	3
Reflux	OESRFX	2	3
Pain	OESPA	3	3
Trouble Swallowing Saliva	OESSV	1	3
Choked When Swallowing	OESCH	1	3
Dry Mouth	OESDM	1	3

Taste Abnormality	OESTA	1	3
Trouble with Coughing	OESCO	1	3
Trouble Talking	OESSP	1	3

Note: The calculation methods and handling of missing data are the same as those shown in Table 4.

9.4 Exploratory Evaluation

To investigate the impact of PD-L1 expression and tumour microenvironment on tumour response and survival, archived baseline tumour tissue samples will be subjected to immunohistochemistry and multiplex immunofluorescence staining using standard protocols.

To explore the potential association between blood biomarkers and treatment efficacy, fresh blood samples will be promptly transported to the laboratory for analysis, where flow cytometry will be used, following the manufacturer's instructions, to detect circulating cytokines and lymphocyte subsets.

10. Statistical Methods

A total sample size of 44 patients is necessary to warrant a power of 80% at a one-sided α level of 0.05 to demonstrate an improvement of 14% in the 1-year survival rate (from 66% in the previous study [RTOG 9405] to 80% in the current study), assuming an accrual period of 18 months and a minimum follow-up period of 12 months. Accounting for a potential dropout rate of up to 10%, the final estimated sample size is 49 patients.

The Kaplan-Meier method will be employed to estimate OS and PFS along with their corresponding 95% confidence intervals (CIs). A log-rank test will be performed to assess survival differences between groups, and Cox proportional hazards regression will be used to estimate hazard ratios (HRs) and 95% CIs. The analysis of efficacy and safety will encompass all patients enrolled in the intention-to-treat (ITT) population during induction therapy and the per-protocol population throughout the entire treatment duration. All reported P values are two-sided with significance levels set at 0.05.

11. Adverse Events

11.1 Definition of Adverse Events

An adverse event refers to the occurrence or worsening of any clinical symptom, syndrome, or disease that occurs during a clinical study and affects the health of the patient. Adverse events may include: new diseases; worsening of symptoms or signs; worsening of concomitant diseases; the influence of test methods or drugs; or a combination of one or more factors.

Any adverse medical event that occurs between the time the patient signs the informed consent and is enrolled in the study and the last visit is considered an adverse event.

Adverse events include but are not limited to:

1) All adverse drug reactions;

2) Obviously unrelated diseases, including new diseases and exacerbations of pre-existing diseases;

3) Injuries and accidents.

11.2 Criteria for the Severity of Adverse Events

The criteria for the severity of adverse events are as follows:

1) Mild: Tolerable to the patient, does not affect treatment or follow-up, does not require specific treatment, and has no impact on the rehabilitation of the patient.

2) Moderate: Unbearable to the patient, requiring specific treatment, which has a direct impact on the rehabilitation of the patient.

3) Severe: Life-threatening, causing death or disability, and requiring emergency treatment.

11.3 Recording of Adverse Events

If serious adverse events (SAEs) occur during the trial, the investigator must report them to the department responsible for clinical research and the ethics committee within 24 hours or no later than the second working day. The researcher must sign and date the report. When, how, and to whom a serious adverse event was reported should be recorded in the original data. The research institutes shall ensure that all reporting procedures required by laws and regulations are implemented.

11.4 Management of Adverse Events

When adverse events are found, researchers can provide necessary treatment based on the condition. All adverse events should be tracked and investigated, and the treatment process and results should be recorded in detail until they are properly resolved or the condition is stable. If the laboratory examination is abnormal, it should be tracked until it returns to normal. Follow-up can be conducted in the hospital, outpatient department, home visit, telephone, and other forms according to the severity of adverse events. Serious adverse events (including abnormal laboratory tests) that were not resolved at the end of the study or the time the patient dropped out early must be followed up to any of the following conditions: 1) event resolved; 2) event stabilized; 3) event returned to baseline; 4) it is determined that the study treatment or participation is not the cause; and 5) when additional information is not available (patient refuses to provide additional information or remains lost to follow-up).

11.5 Management of Serious Adverse Events

A serious adverse event (SAE) is an unexpected medical event occurring during the study period that results in death, life-threatening, hospitalization or prolonged hospitalization, persistent or severe disability, congenital abnormalities/defects and other serious events.

After entering the study, if the patient experiences serious adverse events, in addition to treatment or rescue, the patient should inform the leader of the clinical study, the clinical supervisor, and the ethics committee by telephone or fax within 24 hours of being informed. For all serious adverse events, the investigator should immediately take adequate measures and draft a detailed report of the serious adverse event to be submitted to the relevant administrative authorities and the ethics committee. In the case of death related to treatment, the clinical trial for this group should be stopped immediately, and the ethics committee of the clinical research institution should be informed as soon as possible. Detailed records should be kept, and relevant information should be properly stored.

11.6 Management of Tumor Recurrence and Metastasis

Patients with tumor recurrence and metastasis during follow-up will be recorded in detail in the CRF and treated according to the current clinical pathway.

12. Others

12.1 Informed Consent

Before patient recruitment, the investigator should completely and comprehensively explain the objective of this study, the characteristics of the drugs, and the potential toxicity and risks of the treatment, and ensure that the patients are aware of their rights, risks, and benefits. The informed consent form should be signed before recruitment and preserved in files as paper documentation.

12.2 Case Report

During the study, all patients are required to fill in the case report form according to the study schedule and requirements.

12.3 Ethical Requirements

This study will be conducted according to the Declaration of Helsinki (2000), Good Clinical Practice (GCP) published by China Food and Drug Administration (CFDA), and other relevant regulations.

Before the initiation of the clinical trial, the protocol shall be signed by the investigator, and the trial protocol shall be reviewed and approved by the ethics committee before it can be implemented.

During the trial period, if problems occur in the actual implementation of the clinical trial and the plan needs to be revised, the revised trial protocol shall be approved by the ethics committee again before it can be implemented. Any serious adverse events and deaths that occur during the trial should be reported by the investigator to the ethics committee.

The ethics committee should be informed at the end of the study.

12.4 Quality Control

Investigators should adopt standard operating procedures to ensure the quality control of clinical trials and the implementation of quality assurance systems. All observations and findings in clinical trials should be verified to ensure the reliability of data and to ensure that conclusions in clinical trials are derived from original data. Quality control must be applied at every stage of data processing to ensure that all data are reliable and processed correctly.

12.5 Training of Researchers

Prior to the initiation of clinical trials, the investigator shall be trained according to the trial protocol so that the investigator can understand and be familiar with the nature, procedures, functions, and safety of the trial.

12.6 Improvement of Patients' Compliance

The researcher should carefully implement informed consent so that the patients can fully understand the requirements and cooperate with the investigator. Regular follow-up is necessary to monitor patient compliance. Follow-up should be intensified for those with poor compliance.

12.7 Management of Data

All data will be stored for five years after the termination of the clinical trial.

All information related to this study (including but not limited to the following documents: study protocol, Investigator's Brochure, and summary report) must be kept strictly confidential. Information related to the study or conclusions drawn from the

study may be published by the investigator only with the written consent of the project leader. The researcher should send the paper, abstract, or poster intended for publication or academic lecture to the project leader, who will reply within one month.

Every effort will be made to protect the personal privacy of all subjects. Research-related documents, study reports, publications, and any other publicly disclosed materials shall not include the names or other personal privacy information of the subjects unless required by law. The collection, transmission, processing, and storage of subject information will comply with relevant laws and regulations to ensure that the personal data of subjects is not leaked.

12.8 Study Significance

This study aims to explore the efficacy and safety of induction chemotherapy plus camrelizumab followed by concurrent chemoradiotherapy in patients with unresectable locally advanced esophageal squamous cell carcinoma. The findings are expected to provide important evidence for further Phase III clinical trials and have the potential to change the current treatment strategy for newly diagnosed locally advanced esophageal squamous cell carcinoma.

13. References

1. Bray, F. et al. Global cancer statistics 2018: GLOBOCAN estimates of incidence and mortality worldwide for 36 cancers in 185 countries. *CA Cancer J. Clin.* **68**, 394-424 (2018).
2. Chen, W. et al. Cancer statistics in China, 2015. *CA Cancer J. Clin.* **66**, 115-132 (2016).
3. Arnold, M., Soerjomataram, I., Ferlay, J. & Forman, D. Global incidence of oesophageal cancer by histological subtype in 2012. *Gut* **64**, 381-387 (2015).
4. Zeng, H. et al. Esophageal cancer statistics in China, 2011: Estimates based on 177 cancer registries. *Thorac. Cancer* **7**, 232-237 (2016).
5. Abnet, C. C., Arnold, M. & Wei, W. Q. Epidemiology of Esophageal Squamous Cell Carcinoma. *Gastroenterology* **154**, 360-373 (2018).
6. Cooper, J. S. et al. Chemoradiotherapy of locally advanced esophageal cancer: long-term follow-up of a prospective randomized trial (RTOG 85-01). Radiation Therapy Oncology Group. *JAMA* **281**, 1623-1627 (1999).
7. Minsky, B. D. et al. INT 0123 (Radiation Therapy Oncology Group 94-05) phase III trial of combined-modality therapy for esophageal cancer: high-dose versus standard-dose radiation therapy. *J. Clin. Oncol.* **20**, 1167-1174 (2002).
8. Conroy, T. et al. Definitive chemoradiotherapy with FOLFOX versus fluorouracil and cisplatin in patients with oesophageal cancer (PRODIGE5/ACCORD17): final results of a randomised, phase 2/3 trial. *Lancet Oncol.* **15**, 305-314 (2014).
9. Lordick, F., Mariette, C., Haustermans, K., Obermannová, R. & Arnold, D. Oesophageal cancer: ESMO Clinical Practice Guidelines for diagnosis, treatment and follow-up. *Ann. Oncol.* **27**, v50-v57 (2016).
10. Sanmamed, M. F. & Chen, L. A Paradigm Shift in Cancer Immunotherapy: From Enhancement to Normalization. *Cell* **175**, 313-326 (2018).
11. Sharpe, A. H. & Freeman, G. J. The B7-CD28 superfamily. *Nat. Rev. Immunol.* **2**, 116-126 (2002).
12. Freeman, G. J. et al. Engagement of the PD-1 immunoinhibitory receptor by a novel B7 family member leads to negative regulation of lymphocyte activation. *J. Exp. Med.* **192**, 1027-1034 (2000).
13. Keir, M. E., Butte, M. J., Freeman, G. J. & Sharpe, A. H. PD-1 and its ligands in tolerance and immunity. *Annu. Rev. Immunol.* **26**, 677-704 (2008).
14. Derks, S. et al. Epithelial PD-L2 Expression Marks Barrett's Esophagus and Esophageal Adenocarcinoma. *Cancer Immunol. Res.* **3**, 1123-1129 (2015).
15. Huang, H., Zhang, G., Li, G., Ma, H. & Zhang, X. Circulating CD14(+)HLA-DR(-/low) myeloid-derived suppressor cell is an indicator of poor prognosis in patients with ESCC. *Tumour Biol.* **36**, 7987-7996 (2015).
16. Ohigashi, Y. et al. Clinical significance of programmed death-1 ligand-1 and programmed death-1 ligand-2 expression in human esophageal cancer. *Clin. Cancer Res.* **11**, 2947-2953 (2005).
17. Yagi, T. et al. PD-L1 Expression, Tumor-infiltrating Lymphocytes, and Clinical Outcome in Patients With Surgically Resected Esophageal Cancer. *Ann. Surg.* **269**,

471-478 (2019).
18. Ramakrishnan, R. et al. Chemotherapy enhances tumor cell susceptibility to CTL-mediated killing during cancer immunotherapy in mice. *J. Clin. Invest.* **120**, 1111-1124 (2010).
19. Galluzzi, L., Zitvogel, L. & Kroemer, G. Immunological Mechanisms Underneath the Efficacy of Cancer Therapy. *Cancer Immunol. Res.* **4**, 895-902 (2016).
20. Wan, S. et al. Chemotherapeutics and radiation stimulate MHC class I expression through elevated interferon-beta signaling in breast cancer cells. *PLoS One* **7**, e32542 (2012).
21. Rodriguez-Ruiz, M. E., Vitale, I., Harrington, K. J., Melero, I. & Galluzzi, L. Immunological impact of cell death signaling driven by radiation on the tumor microenvironment. *Nat. Immunol.* **21**, 120-134 (2020).
22. Bracci, L., Schiavoni, G., Sistigu, A. & Belardelli, F. Immune-based mechanisms of cytotoxic chemotherapy: implications for the design of novel and rationale-based combined treatments against cancer. *Cell Death Differ.* **21**, 15-25 (2014).
23. Peng, J. et al. Chemotherapy Induces Programmed Cell Death-Ligand 1 Overexpression via the Nuclear Factor-κB to Foster an Immunosuppressive Tumor Microenvironment in Ovarian Cancer. *Cancer Res.* **75**, 5034-5045 (2015).
24. Kang, Y. K. et al. Nivolumab in patients with advanced gastric or gastro-oesophageal junction cancer refractory to, or intolerant of, at least two previous chemotherapy regimens (ONO-4538-12, ATTRACTION-2): a randomised, double-blind, placebo-controlled, phase 3 trial. *Lancet* **390**, 2461-2471 (2017).
25. Kudo, T. et al. Nivolumab treatment for oesophageal squamous-cell carcinoma: an open-label, multicentre, phase 2 trial. *Lancet Oncol.* **18**, 631-639 (2017).
26. Janjigian, Y. Y. et al. CheckMate-032 Study: Efficacy and Safety of Nivolumab and Nivolumab Plus Ipilimumab in Patients With Metastatic Esophagogastric Cancer. *J. Clin. Oncol.* **36**, 2836-2844 (2018).
27. Doi, T. et al. Safety and Antitumor Activity of the Anti-Programmed Death-1 Antibody Pembrolizumab in Patients With Advanced Esophageal Carcinoma. *J. Clin. Oncol.* **36**, 61-67 (2018).
28. Fuchs, C. S. et al. Safety and Efficacy of Pembrolizumab Monotherapy in Patients With Previously Treated Advanced Gastric and Gastroesophageal Junction Cancer: Phase 2 Clinical KEYNOTE-059 Trial. *JAMA Oncol.* **4**, e180013 (2018).
29. Kato, K. et al. Nivolumab versus chemotherapy in patients with advanced oesophageal squamous cell carcinoma refractory or intolerant to previous chemotherapy (ATTRACTION-3): a multicentre, randomised, open-label, phase 3 trial. *Lancet Oncol.* **20**, 1506-1517 (2019).
