# Supplementary material for: Induction chemotherapy plus camrelizumab followed by concurrent chemoradiotherapy in unresectable locally advanced esophageal squamous cell carcinoma: a single-arm phase II trial
Source: Nat Commun. 2025 Nov 21;16:10292. doi: 10.1038/s41467-025-65206-z (PMC12638862; doi:10.1038/s41467-025-65206-z)
Supplement: Supplementary file 2 — Reporting Summary [file 41467_2025_65206_MOESM2_ESM.pdf]

nature portfolio

Corresponding author(s): Yong Bao

Last updated by author(s): Jul 1, 2025

Reporting Summary

Nature Portfolio wishes to improve the reproducibility of the work that we publish. This form provides structure for consistency and transparency in reporting. For further information on Nature Portfolio policies, see our Editorial Policies and the Editorial Policy Checklist.

Statistics

For all statistical analyses, confirm that the following items are present in the figure legend, table legend, main text, or Methods section.

n/a	Confirmed	
☐	☒	The exact sample size (n) for each experimental group/condition, given as a discrete number and unit of measurement
☒	☐	A statement on whether measurements were taken from distinct samples or whether the same sample was measured repeatedly
☐	☒	The statistical test(s) used AND whether they are one- or two-sided<br>*Only common tests should be described solely by name; describe more complex techniques in the Methods section.*
☐	☒	A description of all covariates tested
☐	☒	A description of any assumptions or corrections, such as tests of normality and adjustment for multiple comparisons
☐	☒	A full description of the statistical parameters including central tendency (e.g. means) or other basic estimates (e.g. regression coefficient) AND variation (e.g. standard deviation) or associated estimates of uncertainty (e.g. confidence intervals)
☐	☒	For null hypothesis testing, the test statistic (e.g. F, t, r) with confidence intervals, effect sizes, degrees of freedom and P value noted<br>*Give P values as exact values whenever suitable.*
☒	☐	For Bayesian analysis, information on the choice of priors and Markov chain Monte Carlo settings
☒	☐	For hierarchical and complex designs, identification of the appropriate level for tests and full reporting of outcomes
☒	☐	Estimates of effect sizes (e.g. Cohen's d, Pearson's r), indicating how they were calculated

Our web collection on statistics for biologists contains articles on many of the points above.

Software and code

Policy information about availability of computer code

Data collection	Microsoft Excel and SPSS statistics (version 22.0) were used for data collection.
Data analysis	R software (version 4.2.1), SPSS statistics (version 22.0), Microsoft Excel, TissueFAXS platform (TissueGnostics, Vienna, Austria) were used for data analysis and figure generation.

For manuscripts utilizing custom algorithms or software that are central to the research but not yet described in published literature, software must be made available to editors and reviewers. We strongly encourage code deposition in a community repository (e.g. GitHub). See the Nature Portfolio guidelines for submitting code & software for further information.

Data

Policy information about availability of data

All manuscripts must include a data availability statement. This statement should provide the following information, where applicable:

- Accession codes, unique identifiers, or web links for publicly available datasets
- A description of any restrictions on data availability
- For clinical datasets or third party data, please ensure that the statement adheres to our policy

All data requests will undergo review by the First Affiliated Hospital of Sun Yat-Sen University to assess any potential intellectual property or confidentiality obligations. A proposal detailing the study objectives and statistical analysis plan will be required for evaluation. Additional materials may also be requested during the evaluation process. Data will be available upon request 12 months after the publication of this article. Detailed individual data are available under restricted

access for both legal and ethical concerns. Requests for access to de-identified participant data from this study can be submitted via email to baoyong@mail.sysu.edu.cn, accompanied by a detailed proposal for approval. Please allow 1 month for a response to the request. The raw identifying individual participant data are protected and are not available due to data privacy laws. The study protocol is available as Supplementary Note in the Supplementary Information file. The remaining data are available within the Article, Supplementary Information, or Source Data file. Source data are provided with this paper.

Research involving human participants, their data, or biological material

Policy information about studies with human participants or human data. See also policy information about sex, gender (identity/presentation), and sexual orientation and race, ethnicity and racism.

Reporting on sex and gender	Sex was not considered in the study design, and the sex of participants was determined based on self-report. Both male and female participants who met the inclusion/exclusion criteria were eligible for this study. The results of the study apply to both male and female participants. No sex analysis was therefore carried out. The sex distribution was 41 men (83.7%) and 8 women (16.3%).
Reporting on race, ethnicity, or other socially relevant groupings	Race, ethnicity, or other socially relevant groupings were not considered in the research design.
Population characteristics	Between July 12, 2020, and October 14, 2022, a total of 69 patients with esophageal squamous cell carcinoma were screened for eligibility; 49 eligible patients with unrestable locally advanced esophageal squamous cell carcinoma were enrolled. The median age was 62 years (range: 41–74), comprising of 41 males (83.7%) and 8 females (16.3%). At baseline, stage III or IVA disease was diagnosed in 28 patients (57.1%), while 16 patients (32.7%) had stage IVB disease solely due to supraclavicular lymph node metastasis.
Recruitment	Patients were offered the opportunity to participate in the clinical trial by investigators during routine visits at the First Affiliated Hospital of Sun Yat-Sen University. All patients who met the criteria were included in the study, with no potential for self-selection bias. Investigators at the First Affiliated Hospital of Sun Yat-Sen University screened and enrolled participants who met all the inclusion criteria and none of the exclusion criteria as defined in the protocol. All participants provided written informed consent prior to enrollment in the study.
Ethics oversight	The study was conducted in accordance with the Declaration of Helsinki and Good Clinical Practice guidelines and was approved by the Ethics Committee of the Guangdong Association Study of Thoracic Oncology. All participants provided written informed consent prior to enrollment. The trial is registered at the Chinese Clinical Trial Registry (ChiCTR.org.cn) under the identifier ChiCTR2000034304.

Note that full information on the approval of the study protocol must also be provided in the manuscript.

Field-specific reporting

Please select the one below that is the best fit for your research. If you are not sure, read the appropriate sections before making your selection.

☒ Life sciences ☐ Behavioural & social sciences ☐ Ecological, evolutionary & environmental sciences

For a reference copy of the document with all sections, see nature.com/documents/nr-reporting-summary-flat.pdf

Life sciences study design

All studies must disclose on these points even when the disclosure is negative.

Sample size	A total of 44 patients were needed to observe an improvement of 14% in 1-year OS rate (from 66% in the previous study to 80% in the current study), with a one-sided α level of 0.05, power of 80%, an accrual period of 18 months, and a minimum follow-up period of 12 months. Assuming a dropout rate of 10%, the final estimated sample size was determined as 49 patients.
Data exclusions	Between July 12, 2020, and October 14, 2022, 69 patients with esophageal squamous cell carcinoma (ESCC) were screened. Twenty patients (29%) were excluded during screening due to failure to meet protocol-defined inclusion criteria. 49 patients initiated the protocol-specified treatment. During the study period, 3 participants (6%) withdrew. All patients enrolled in this study were included for analysis. Withdrawn patients were excluded from the per-protocol analysis but retained in the intention-to-treat (ITT) population.
Replication	This prospective, single-arm, phase 2 trial (ChiCTR2000034304) achieved the study's predefined endpoint. An exploratory post-hoc analysis was conducted to assess the robustness of the primary findings. Propensity-score matching (PSM) was applied to compare the trial group comprising 46 patients from the per-protocol set (PPS) receiving the experimental intervention with the control group of 71 patients treated with standard care. The experimental group demonstrated significantly improved survival compared to the matched controls.
Randomization	This study was a prospective, single-arm, phase 2 trial (ChiCTR2000034304) without a control arm, but an exploratory post-hoc analysis was conducted to assess the robustness of the primary findings.
Blinding	This study was a prospective, single-arm, phase 2 trial (ChiCTR2000034304) without a control arm. Given that there was no randomization, and patients were included in one consecutive cohort, the blinding did not apply.

Reporting for specific materials, systems and methods

We require information from authors about some types of materials, experimental systems and methods used in many studies. Here, indicate whether each material, system or method listed is relevant to your study. If you are not sure if a list item applies to your research, read the appropriate section before selecting a response.

Materials & experimental systems

n/a	Involved in the study
☐	☒ Antibodies
☒	☐ Eukaryotic cell lines
☒	☐ Palaeontology and archaeology
☒	☐ Animals and other organisms
☐	☒ Clinical data
☒	☐ Dual use research of concern
☒	☐ Plants

Methods

n/a	Involved in the study
☒	☐ ChIP-seq
☐	☒ Flow cytometry
☒	☐ MRI-based neuroimaging

Antibodies

Antibodies used	Camrelizumab (SHR-1210), PD-L1 immunohistochemistry assay (clone 22C3; DAKO Autostainer Link48; RTU) , Anti-CD8 (Cat# ab237709, Abcam), Anti-CD4 (Cat# ab133616, Abcam), Anti-CCR7 (Cat#ab253187, Abcam), Anti-DR5 (Cat# ab8416, Abcam), Anti-pan-CK (Cat# Kit-0009, MXB Biotechnologies).
Validation	No new antibodies were generated in this study. All antibodies are commercially available with detailed descriptions available in the manufacturer's websites.

Clinical data

Policy information about clinical studies
All manuscripts should comply with the ICMJE guidelines for publication of clinical research and a completed CONSORT checklist must be included with all submissions.

Clinical trial registration	The trial is registered at the Chinese Clinical Trial Registry (ChiCTR.org.cn) under the identifier ChiCTR2000034304.
Study protocol	The study protocol is available in Supplementary Materials.
Data collection	Participants were consecutively recruited from the First Affiliated Hospital of Sun Yat-Sen University (Guangzhou, China) between July 12, 2020, and October 14, 2022. Data were collected using Microsoft Excel and SPSS Statistics 22.0 at the First Affiliated Hospital of Sun Yat-Sen University. Follow-up is ongoing.
Outcomes	The endpoints of the study are as follows:<br>1) Primary endpoint: To evaluate the 1-year survival rate of patients with unresectable locally advanced esophageal squamous cell carcinoma treated with camrelizumab combined with induction chemotherapy followed by concurrent chemoradiotherapy;<br>2) Secondary endpoint: To evaluate overall survival (OS), progression-free survival (PFS), objective response rate (ORR), disease control rate (DCR), duration of response (DoR), safety, and health-related quality of life (EORTC QLQ-C30, EORTC QLQ-OES18);<br>3) Exploratory endpoint: To investigate the potential association between tumor tissue and/or blood biomarkers and treatment efficacy.

Plants

Seed stocks	The study did not involve any plants.
Novel plant genotypes	Not applicable.
Authentication	Not applicable.

Flow Cytometry

Plots

Confirm that:

☒ The axis labels state the marker and fluorochrome used (e.g. CD4-FITC).

☒ The axis scales are clearly visible. Include numbers along axes only for bottom left plot of group (a 'group' is an analysis of identical markers).

☒ All plots are contour plots with outliers or pseudocolor plots.

☒ A numerical value for number of cells or percentage (with statistics) is provided.

Methodology

Sample preparation	The patient blood samples were collected in 10ml EDTA tubes and immediately centrifuged at 1500rpm for 30 minutes at 4ºC without brake.
Instrument	We employed the BD FACSCanto II flow cytometer (Becton Dickinson) to analyze lymphocyte subpopulations and T helper 1 (Th1)/Th2 cytokines in the venous blood anticoagulated with EDTA, following the manufacturer's instructions. The lymphocyte subpopulation analysis was performed using a BD Multitest 6-color TBNK kit (Becton Dickinson), which included total T lymphocytes (CD3+ CD19- T lymphocytes), helper T lymphocytes (CD3+CD4+ T lymphocytes), cytotoxic T lymphocytes (CD3+CD8+ T lymphocytes), B lymphocytes (CD3-CD19+ B lymphocytes), and natural killer (NK) cells (CD3-CD16+ CD56+ NK cells). The cytokine analysis was conducted using a Human Th1/Th2 subgroup detection kit from Hangzhou Saikey Biotechnology Co., LTD., which included interleukin (IL)-2, IL-4, IL-6, IL-10, tumor necrosis factor(TNF)-α, and interferon-gamma(IFN-γ).
Software	The flow cytometry data were analyzed using BD FACSCanto™ Clinical Software, which automatically calculates lymphocyte subset percentages and absolute counts when BD Trucount™ Tubes are employed.
Cell population abundance	BD Multitest™ 6-Color TBNK Reagent, with optional BD Trucount™ Tubes, was used in conjunction with BD FACSCanto™ II flow cytometers to determine the percentages and absolute counts of mature human lymphocyte subsets in peripheral whole blood for immunophenotyping.
Gating strategy	Visual validation ensures distinct clusters (e.g., lymphocytes vs monocytes/granulocytes). Fluorescence triggering minimizes contamination from unlysed RBCs. Avoid FSC thresholding with BD Trucount™ Tubes. Controls (e.g., BD Multi-Check™) verify gating accuracy. This hierarchical approach ensures precise quantification of lymphocyte subsets (T, B, NK) and their functional populations ($CD4^+/CD8^+$), critical for immunophenotyping in clinical diagnostics.

☐ Tick this box to confirm that a figure exemplifying the gating strategy is provided in the Supplementary Information.
